# Enhancing free space optical CubeSat (FSOCS) communication links using low-complexity LDPC decoding techniques

**DOI:** 10.1038/s41598-026-68721-1

**Published:** 2026-09-09

**Authors:** Albashir A. Youssef, Yahya Z. Mohasseb

**Affiliations:** Electronics and Communication Department, Arab Academy for Science, Technology and Maritime Transport, Cairo, 2033 Egypt

**Keywords:** LDPC, FSO, Cirrus, Engineering, Mathematics and computing

## Abstract

In this work, a low-complexity, low-power, LDPC decoding scheme is presented to improve the performance of Free-Space Optical CubeSat (FSOCS) communication links. This hard-decision bit-flipping scheme aims to improve the performance–complexity tradeoff compared to existing LDPC decoding techniques. Our simulations, which span a wide variety of FSOCS channel models including inter-satellite links and atmospheric optical channels, demonstrate that the proposed decoder approaches the performance of the soft-decision Min-Sum decoder while requiring fewer iterations compared to state-of-the-art hard-decoding schemes. The reduced computational complexity and decoding time make it suitable for resource-constrained CubeSat platforms.

## Introduction

The rapid expansion of NewSpace activities has renewed interest in CubeSat platforms as low-cost, rapidly deployable solutions for space missions^1,2^. As CubeSats evolve from educational platforms into viable spacecraft supporting Earth observation, scientific, and communication missions^3^, their communication systems are becoming an active area of research. In particular, high-data-rate links operating under stringent size, weight, and power constraints are necessary to support such missions^4^.

Free-space optical CubeSat systems (FSOCS) have emerged as a promising solution for high-data-rate inter-satellite communication links^5^. Several CubeSat platforms have been designed specifically for inter-FSOCS communication networks, for example,^6^ and^7^. FSOCS links introduce several technical challenges that can degrade communication reliability, creating several interesting research areas. These areas include accurate acquisition and tracking of the optical beam^8^ as well as combating atmospheric turbulence-induced scintillation and fluctuations in received optical power^9^ and turbulence-induced fading^10^.

As multi-satellite networks transition toward dense, autonomous multi-CubeSat constellations, the demand for adaptive communication and distributed resource management becomes critical^11^. In distributed satellite swarms, collaborative tasks require nodes to dynamically balance processing overhead and transmission media constraints to minimize end-to-end latency. For instance, recent strategies explore joint communication-media selection and bandwidth allocation (e.g., trading off token-based vs. key-value cache-based transmission frameworks) to optimize multi-agent collaboration over heterogeneous links. To support such high-level, adaptive intelligence at the network tier, the underlying physical layer must deliver highly efficient, low-latency error correction. This highlights the necessity for low-complexity, hard-decision LDPC decoders capable of seamlessly operating under dynamic link conditions without imposing heavy computing footprints on resource-constrained satellite nodes.

Error-control coding techniques for CubeSat communication channels have been investigated in several studies. A comprehensive survey in^12^ evaluates modulation and coding techniques for CubeSat communications, including Reed-Solomon and convolutional coding schemes. Additional empirical evaluation in^13^ shows that combinations of Reed-Solomon and convolutional coding improve bit-error-rate (BER) performance under different CubeSat channel models. These studies suggest that more powerful coding techniques, like Low-Density Parity Check Codes (LDPC), should be investigated for future CubeSat communication systems.

Indeed,^1^ identifies LDPC coding as a promising approach to enhance communication performance and reduce the required transmit power. LDPC coding is also adopted in practical CubeSat communication system designs, such as the proprietary broadband system described in^14^. As with most CubeSat communication systems employing LDPC codes, Krenz et al.^14^ relies on a soft-decision decoding technique called belief-propagation decoding. Similarly, the decoding approach proposed in^15^ resembles belief-propagation decoding and therefore suffers from high computational complexity. The baseband modem design presented in^16^ also employs belief-propagation LDPC decoding, which is associated with significant hardware complexity.

While hard-decision decoding variants like Weighted Bit-Flipping (WBF), Implementation Efficient Reliability Ratio (IERR), and Low Complexity IERR (LCRR) offer reduced computational complexity, they face distinct limitations in dynamic environments. WBF uniformly penalizes all connected variable nodes, IERR introduces significant processing delays via global ratio matrices, and LCRR merely halts decoding once oscillations occur rather than actively preventing them. Furthermore, conventional bootstrap-based bit-flipping relies on offline thresholding parameters calibrated to static channel models, rendering them vulnerable to severe Free-Space Optical (FSO) atmospheric turbulence. To overcome these challenges, this paper proposes a dynamic, syndrome-driven bootstrap architecture that eliminates offline thresholds, lowers iteration times, and directly mitigates cyclic error oscillations.

In this paper, a low-complexity LDPC hard-decision decoding scheme is proposed for FSOCS communication links. The proposed decoder is designed to address the stringent computational and energy constraints of CubeSat platforms while maintaining reliable communication performance over FSOCS channels. The proposed technique aims to improve the performance–complexity trade-off ofg bit-flipping decoding techniques^17–19^. Simulation results across representative FSOCS channel models, including inter-satellite links and atmospheric optical links under cirrus and thin-cirrus conditions, demonstrate that the proposed decoder consistently achieves lower BERs than existing hard-decision decoding techniques while requiring fewer decoding iterations. The proposed scheme approaches the error-rate performance of the soft-decision Min-Sum decoder while maintaining substantially lower computational complexity and decoding time. Although the decoding time of the proposed method is slightly higher than that of the fastest hard-decision decoder considered, the difference remains moderate while providing a significant improvement in error-rate performance. These characteristics make the proposed decoder particularly suitable for resource-constrained CubeSat communication systems.

The main contributions achieved by this work is a fully dynamic, online LDPC decoding architecture: Unlike conventional bootstrap methods that rely on static, offline threshold calibrations (*γ *), the primary contribution of this work is a novel paradigm that completely eliminates offline threshold selection. We achieve this by introducing a joint mechanism consisting of:

1. Unreliable-check-node selection: an online syndrome-driven mechanism ($s = zH^T$) that isolates faulty checks on-the-fly without channel state information.
2. Soft-value update rule: a localized scaling approach targeting exclusively the minimum soft value $\vert {}y_{n_{min}}\vert {} = \min (\vert {}y_n\vert {})$ per violated node, drastically reducing the time per iteration.

The remainder of this paper is organized as follows: “The FSOC system model and associated channel models” presents the FSOCS system model and describes the channel models considered, including inter-satellite optical links and atmospheric optical channels under different turbulence conditions. “LDPC encoding and decoding techniques” provides an overview of the LDPC coding framework and introduces the decoding techniques considered in this study. “Proposed low-complexity LDPC decoding scheme” describes the proposed low-complexity LDPC decoding technique and presents an analysis of its computational complexity. “Results” presents simulation results and performance evaluations for the considered FSOCS channel models, including comparisons of bit error rate, decoding iterations, and decoding time. Finally, the discussion is presented.

**Fig. 1:**
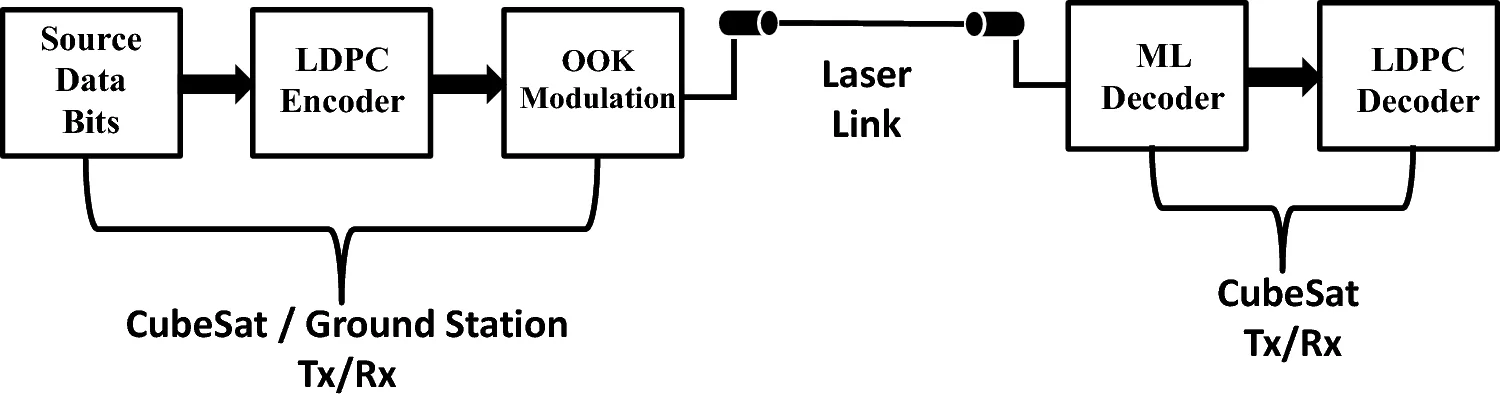
FSOCS-to-FSOCS/ground station laser communication links.

## The FSOC system model and associated channel models

The system model of FSOCSs considered in this work is shown in Fig. 1 and illustrates how FSOCSs communicate with each other or with the ground station. In Fig. 1. The source data is LDPC-encoded at the transmitter, either on a CubeSat or at a ground station, depending on the communication type if it is inter-FSOCS or Uplink/Downlink from FSOCS to ground station. Then, at the receiver, the received coded data is decoded from another CubeSat or a ground station.

### Inter-FSOCS links channel model

In the simulations, the power model is used as the channel model for inter-FSOCS links. The inter-FSOCS power model is determined using the following equation^20^:

1 $$\begin{aligned} p_{r} = p_{t}\,(G_{r}\,G_{t}\,l_{r}\,l_{t}\,l_{p}) \,\eta _{r}\,\eta _{t} \end{aligned}$$

where $p_{t}$ and $p_{r}$ are the transmitting power and the receiving power in watts of the inter-FSOCS link. Also, 2 and 3 are the transmission and receiving gains, defined by the following equations^20^:

2 $$\begin{aligned} G_{t} = \frac{16}{(\theta _{t})^{2}} \end{aligned}$$

3 $$\begin{aligned} G_{r} = \frac{(\pi D_{r})}{\lambda ^{2}} \end{aligned}$$

Also, $\theta _{t}$ is the divergence angle of the transmitter, and $D_{r}$ represents the telescope diameter of the receiver. The pointing losses in the transmitter and receiver are as follows^20^:

4 $$\begin{aligned} l_{t} = \exp {(-G_{t}\theta _{t}^{2})} \end{aligned}$$

5 $$\begin{aligned} l{r} = \exp {(-G_{r}\theta _{r}^{2})} \end{aligned}$$

For the loss imposed by the free space path, it is termed as follows^20^:

6 $$\begin{aligned} l{p} = (\frac{\lambda }{4\pi d_{ss}})^{2} \end{aligned}$$

Where $d_{ss}$ represents the distance of propagation within FSOCSs, also, *λ * is the wavelength.

### Channel model within ground station and FSOCS

Geometrical and Mie scattering are considered in terms of optical uplink $l_{a_{up}}$ and downlink $l_{a_{down}}$ atmospheric attenuations. The primary reason for Mie scattering is the presence of water particles in the channel. Also, it causes redirection of signals in its direction, whereas reflection, refraction, and scattering of the optical signal are due to geometrical scattering. The reception power $P_{r}$ is as follows^21^:

7 $$\begin{aligned} P_{r} = P_{t}\,G_{t}\,G_{r}\,l_{t}\,l_{r}\,l_{a}\,l_{pg}\,\eta _{t}\,\eta _{r} \end{aligned}$$

where $l_{pg}$ is the free-space loss between FSOCS and the ground station. The other factors are the same as in the inter-FSOCS case. The separation distance within FSOCSs and ground stations is defined as follows^22^:

8 $$\begin{aligned} d_{gs} = R\Bigg (\sqrt{\bigg (\frac{R + h}{R}\bigg )^{2} - (cos(\theta _{e}))^{2}} - sin (\theta _{e})\Bigg ) \end{aligned}$$

Given that $R = r + h_{e}$ and $h = h_{s} - h_{e}$, while *r* is the earth’s radius. The height of the ground station is $h_{e}$, and the altitude of the FSOCS is $h_{s}$. The elevation angle of the ground station is $\theta _{e}$ in degrees. The loss due to free space is denoted by $l_{pg}$ and expressed as follows:

9 $$\begin{aligned} l_{pg} = \bigg (\frac{\lambda }{4\pi d_{gs}}\bigg )^{2} \end{aligned}$$

Mie scattering’s attenuation occurs when the atmospheric particle diameter is larger than or equal to the laser beam wavelength. Besides, the non-uniformity of the laser beam’s phase causes the transmitted beam to deviate from its intended path, and water particles are present at lower atmospheric levels. Mie scattering effect is expressed as follows^23^:

10 $$\begin{aligned} \gamma = A(h_{e})^{3} + B (h_{e})^{2} + C h_{e} + D \end{aligned}$$

where *γ * represents the ratio of extinction. The empirical coefficients *A*, *B*, and *C* mainly depend on the wavelength of the laser beam. They are expressed as follows:

11 $$\begin{aligned} A = 545e-6 \lambda ^{2} + 2e^{-3} \lambda - 3.8e^{-3} \end{aligned}$$

12 $$\begin{aligned} B = 6.28e^{-3} \lambda ^{2} - 23e-3 \lambda +4.39e^{-3} \end{aligned}$$

13 $$\begin{aligned} C = -28e-3 \lambda ^{2} + 101e-3 \lambda - 180e^{-3} \end{aligned}$$

14 $$\begin{aligned} D = -228e^{-3} \lambda ^{3} + 922e^{-2} \lambda ^{2} - 1.26\lambda + 719e^{-3} \end{aligned}$$

Therefore, atmospheric attenuation resultant from Mie scattering is determined as follows:

15 $$\begin{aligned} i_{M} = e^{\frac{-\gamma }{sin(\theta _{e})}} \end{aligned}$$

The next impairment arises in the channel model, concerning the ground station-to-FSOCS link, where geometric scattering occurs at the Earth’s surface. Its main causes are fog, dense clouds, water molecules, and rain. The factor that affects the channel by geometrical scattering is the visibility (*v*), which is calculated as follows^24^:

16 $$\begin{aligned} v = \bigg (\frac{1.002}{nl_{w}}\bigg )^{0.6473} \end{aligned}$$

The visibility *v* is determined in km, while *n* is the cloud number concentration is calculated by $cm^{3}$ and $l_{w}$ represents the liquid’s content $g/m^{-3}$ and $\theta _{a}$ represents the coefficient of attenuation, which is determined by^24^:

17 $$\begin{aligned} \theta _{a} = \frac{3.91}{v} (\frac{\lambda }{550})^{-\phi } \end{aligned}$$

The coefficient of particle size is related to Kim’s model, while the law of Beer-Lambert is expressed as^25^:

18 $$\begin{aligned} i_{x} = e^{-\mu x} \end{aligned}$$

Where *μ * is the attenuation-dependent coefficient in terms of *x*, which is the transmission path distance.

The geometrical scattering, which causes atmospheric attenuation, can be determined by maintaining the law of Beer-Lambert as follows:

19 $$\begin{aligned} i_{G} = e^{-\theta _{a}\, d_{a}} \end{aligned}$$

The distance traveled by the laser beam through the troposphere is $d_{a}$. It is calculated as^26^:

20 $$\begin{aligned} d_{a} = (h_{a} - h_{e}) \,csc(\theta _{e}) \end{aligned}$$

where, $h_{a}$ represents the troposphere’s height in km.

In the case of the uplink communication link, the geometrical scattering is the main channel impairment, while for the downlink communication link, both Mie and geometrical scattering are considered, and they are calculated as follows^27^:

21 $$\begin{aligned} l_{a_{up}} = i_{G} = e^{-\theta _{e}\, d_{a}} \end{aligned}$$

22 $$\begin{aligned} l_{a_{down}} = i_{G}\, i_{M} = e^{-\theta _{e} \, d_{a}}\, e^{-\theta _{a} \,d_{a}} \end{aligned}$$

### Atmospheric Turbulence in the FSOCS Communication Link

The atmospheric turbulence modeled by the exponentiated Weibull fading channel is considered for the FSO links between ground stations and FSOCSs. The exponentiated Weibull fading channel has a probability density function (PDF) as^27^:

23 $$\begin{aligned} f(i) = \bigg (\frac{\beta \,\alpha }{\eta }\bigg ) \, \bigg (\frac{i}{\eta })^{\beta - 1}\bigg )\, e^{-\big (\frac{I}{\eta }\big )^{\beta }} \, \bigg (1 - e^{\bigg (-\frac{I}{\eta }\bigg )^{\beta }}\bigg )^{\alpha } \end{aligned}$$

where *β * and *α * are shape parameters. The scale parameter of the ground station is represented by *η *. All these parameters are determined as follows^27^:

24 $$\begin{aligned} {\begin{matrix} \beta = 1.012 (\alpha \sigma _{i}^{2})^{\frac{13}{25}} + \frac{71}{500}\\ \alpha = \frac{\frac{361}{50} \alpha ^{\frac{2}{2}}}{\gamma (2.487 \sigma _{i}^{\frac{1}{3}} - \frac{13}{125}}\\ \eta = \frac{1}{\alpha \Gamma (1 + \frac{1}{\beta })g(\alpha , \beta )} \end{matrix}} \end{aligned}$$

as $g(\alpha , \beta )$ is the function of the dependent constant variable, *α * and *β * the scintillation index is $\sigma _{i}$ for the ground station, which is determined as follows^28^:

25 $$\begin{aligned} \sigma _{i} = exp\Bigg (\frac{51}{100} \frac{\sigma _{r}^{2}}{\bigg (1+\frac{69}{100}\sigma _{r}^{\frac{12}{5}}\bigg )} + \sigma _{i} \bigg (\frac{\frac{49}{100}\sigma _{r}^{2}}{1+\frac{111}{100}\sigma _{r}\frac{12}{5}}\bigg )^{\frac{7}{6}}\Bigg ) -1 \end{aligned}$$

where, $\sigma _{r}$ is the Rytov variance and expressed as^28^:

26 $$\begin{aligned} \sigma _{r} = \frac{9}{4} K^{\frac{7}{6}} sec^{\frac{11}{6}} (\zeta ) \int _{h_{e}}^{h_{s}} C_{n}^{2}(h) (h - h_{e})^{\frac{5}{6}} .dh \end{aligned}$$

while *K* the number of laser beams, the zenith angle *ζ * of the ground station, the satelite altittude is $h_{s}$, and the altitude-dependent refractive index constant $C_{n}^{2}$ are computed as follows^29^:

27 $$\begin{aligned} {\begin{matrix} C_{n}^{2}(H) = 8.148 \times 10^{-56}\, V_{r}^{2} \,H^{10} \,exp\bigg (-\frac{h}{1000}\bigg )\\ + 2.7 \times 10^{-16} \,exp\bigg (-\frac{H}{1500}\bigg ) + c_{o} \,exp\bigg (- \frac{h}{100}\bigg ) m^{-\frac{2}{3}} \end{matrix}} \end{aligned}$$

The term $V_{r}$ represents the r.m.s. speed for the ground wind, and $c_{o}$ is also defined as the nominal value of the ground refractive index constant.

**Fig. 2:**
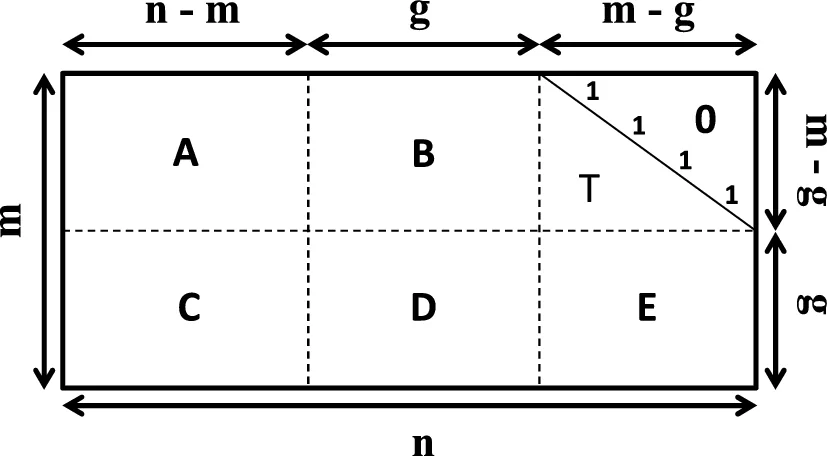
The approximate lower triangular form of the parity-check matrix.

## LDPC encoding and decoding techniques

Sparse parity check matrices ${\mathbf{H}}$ are used to define LDPC codes. Therefore, instead of transforming ${\mathbf{H}}$ into its generator matrix ${\mathbf{G}}$, an efficient encoding approach will be applied where the sparse nature of the ${\mathbf{H}}$ matrix will be altered, resulting in a potentially unreasonable amount of complexity^30^. The encoding technique developed in^30^ was used in the simulations of this work. As shown in Fig. 2, constructing an encoder via Gaussian elimination yields a precise lower-triangular form. The vector ${\mathbf{x}}$ will be decomposed into a parity portion (${\mathbf{p}}$) and a systematic part (${\mathbf{s}}$), producing ${\mathbf{x}} = \mathbf {[s; p]}$. Preprocessing of $O(n^{3})$ operations is needed for the encoding procedure that processes the matrix ${\mathbf{H}}$ into the required shape. Consequently, the matrix will become less sparse.

To accomplish this encoding, it is estimated that it requires approximately $n^{2} D_{r}(1-r)/2$ XOR operations, where *r* is the code rate. For these codes, this encoding is appropriate. Unusually, the encoding method described in^30^ requires quadratic encoding because the constant components preceding the $n^{2}$ term are often insignificant. Blocks with long lengths will still allow an appropriate level of encoding complexity. Fig. 2 shows the LDPC encoder maintained. It can transform the parity-check matrix into the form shown in Fig. 2, which is nearly lower-triangular, provided that permutations are applied only to columns and rows. Additionally, these matrices are primarily distinguished by their sparsity, except for the diagonal of the bottom triangular matrix. Consequently, this matrix will be multiplied from the left by the matrix presented in Fig. 2.

### Weighted bit flipping (WBF) decoding technique

^17^ The only attempt to use LDPC decoding techniques for FSOCS communications is in^17^. The technique maintained in^17^ is identified as a hard decision technique and is termed the “Weighted Bit Flipping” (WBF) technique. The performance of WBF is investigated in^17^. It achieved comparable BER and complexity performance. Identifying the variable nodes that are significantly unreliable associated with each check node is the first step in the WBF decoding process. The following equation defines this step:

28 $$\begin{aligned} \mid y_{n_{min}} \mid =\lbrace \min \mid y_{n} \mid : n \in \mathscr {N}(m)\rbrace \end{aligned}$$

Where $n_{min}$ represents the index of the lesser soft value of variable nodes connected to the check node *m*.The minimum absolute term in the received codeword is calculated as^17^ as $\mid y_{n} \mid$ is the absolute value of $y_{n}$, characterizing the reliability calculation of the received message. Since the binary counterparts $b_{n}$ and $y_{n}$ are their soft value with formidable reliability $\mid y_{n} \mid$. It is for the hard-decision $b_{n}$ digit to be leveled up. Every variable node determination of the error term $E_{n}$ is expressed by:

29 $$\begin{aligned} E_{n} = \sum _{m\in {M(n)}} (2s_{m} - 1) \mid y_{n_{min}} \mid \end{aligned}$$

as the syndrome-associated bit $s_{m}$ belongs to the check node *m*. The $E_{n}$ represents the weight checksum that is connected to the *n* code bit.

The procedures of decoding steps performed by the WBF technique are listed in Technique 1.

**Technique 1:**
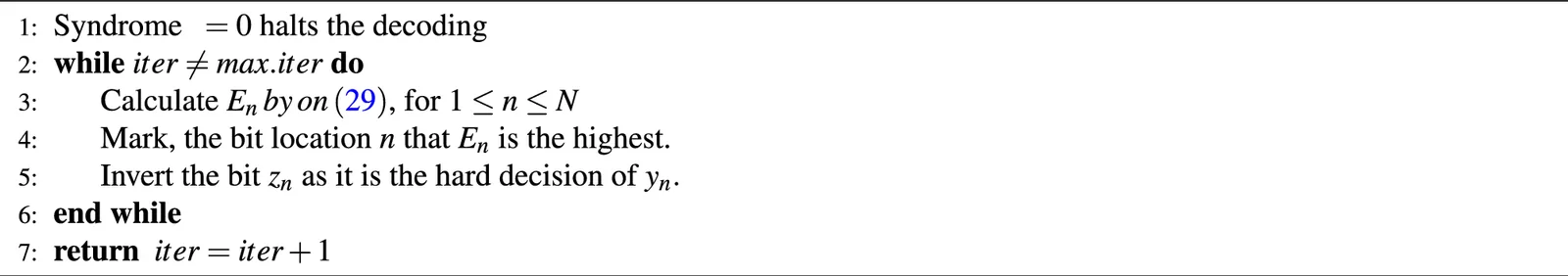
WBF decoder^17^.

### Implementation efficient reliability ratio (IERR) weighted bit flipping decoding

^18^

WBF’s primary weakness is that it treats an unreliable bit as a check violation. All message nodes linked to a check node, however, are accountable for this specific check’s violation. In other words, if the check they participate in is violated, all message nodes may be modified. However, if two distinct message nodes are involved in the same violated parity check, the likelihood that the check is violated because one of the message nodes has a high soft-magnitude is less than the likelihood that the message node has a low soft-magnitude. Therefore, it provided a new metric called the Reliability Ratio (RR), which is defined as follows^31^:

30 $$\begin{aligned} R_{ij} = \alpha \frac{abs(y_{j})}{abs(y_{i}^{max})} \end{aligned}$$

as $y_{i}^{max}$ is the maximum soft value within the check node. Therefore, the error term will be calculated as follows^31^:

31 $$\begin{aligned} E_{j} = \sum _{i \in M(i)} \frac{(2s_{i} - 1)}{R_{ij}} \end{aligned}$$

The Reliability Ratio WBF technique is found to consume a significant number of operations^31^. Therefore,^18^ proposed a crucial adjustment to minimize the complexity while preserving the BER gain compared to WBF. This technique is called Implementation-Efficient Reliability Ratio WBF and will be abbreviated IERR hereafter. Therefore, the proposed technique in^18^ reduces the complexity calculation error term by replacing the reliability ratio factor with $T_{m}$. This term aims to reduce the decoding time required by the RRWBF technique.

**Technique 2:**
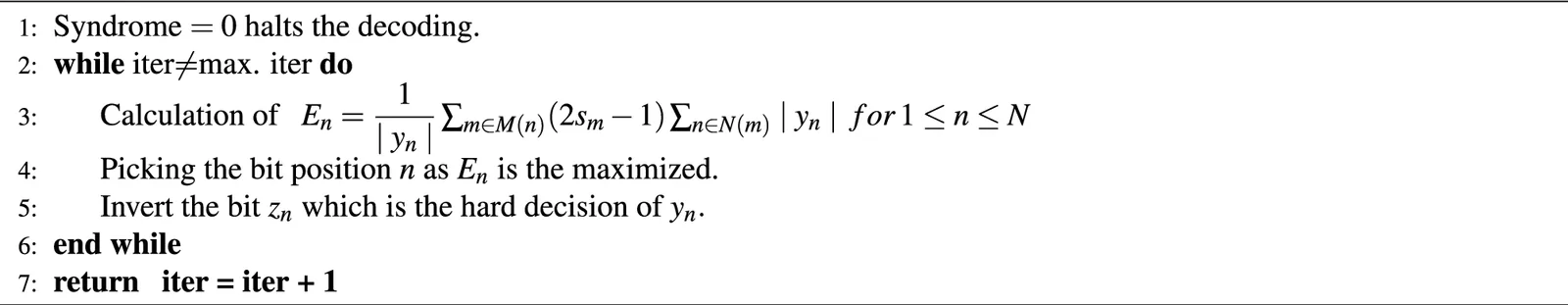
IERR decoder^18^.

**Fig. 3:**
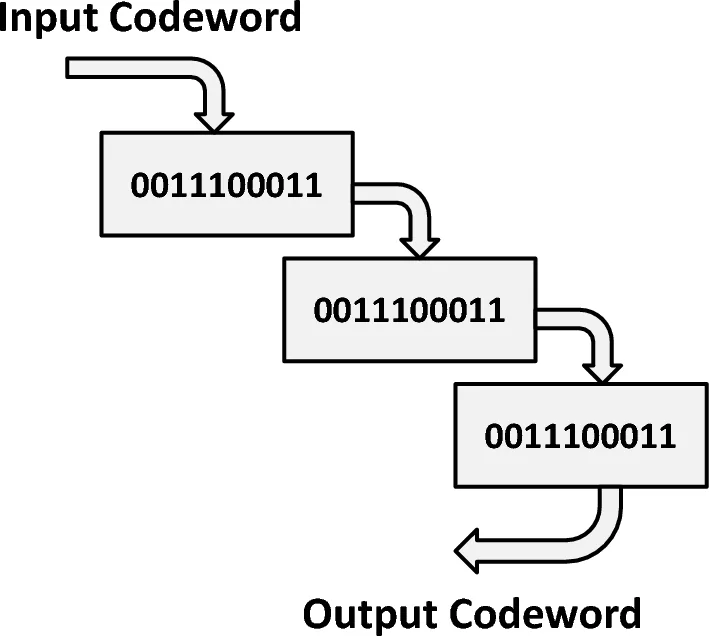
The oscillation phenomenon occurs in the IERR example.

### Low complexity IERR (LCRR) decoding technique^19^

The longer decoding process, particularly at the check-node and variable-node steps, is the primary drawback of the latter iterative decoders. It is the main issue with IERR decoding. There is no further improvement in BER when the number of iterations of the given decoding method increases. Instead, more computational processing time is required per iteration. Furthermore, an oscillation phenomenon occurs, increasing the decoding delay without further improvement in BER^19^.

Sometimes, bit-flipping-based techniques successfully decode the received codeword, primarily at a subordinate $E_{b}/N_{o}$. This is because, in such a case, decoding attempts to make the syndrome vector non-zero, resulting in an unsuccessful decoding of the received codeword. Furthermore, with a considerable number of iterations (more than 1000), the error performance will show no noticeable improvement. It has been observed that at lower $E_{b}/N_{o}$s, the syndrome vector eventually becomes non-zero, and decoding constantly flips, requiring more processing resources without improving BER performance.

The low-complexity version of the IERR includes a decision step to identify the scenarios shown in IERR and to limit the iteration loop by choosing to either continue decoding or terminate the iteration, thereby producing an improved decoded message. In particular, Haddadi et al.^19^ proposed a method for preserving various states that begins with a syndrome check at each iteration. Therefore, Haddadi et al.^19^ included a three-entry register, because if the same bit in the decoded code word is flipped again to revert to the original state, only two iterations are needed to obtain the same syndrome vector, beginning with the initial vector. This low-complexity enhancement will be abbreviated LCRR in the sequel.

This makes it clear that, to identify oscillatory phenomena, three entries are needed; the minimal number of registers required to accumulate incoming binary vectors and perform a correlation between the first and third entries is three flip-flops. The top flip-flop register accumulates each renewed entry, while the remaining entries are shifted down to replace the last input. As a result, once the oscillation is achieved, decoding will stop, and no further performance gains will occur. This additional requirement does not affect the reduction in complexity without compromising the performance of the proposed technique in^19^. For further explanation, the oscillation phenomena discussed in the previous paragraph are illustrated in Fig. 3.

**Technique 3:**
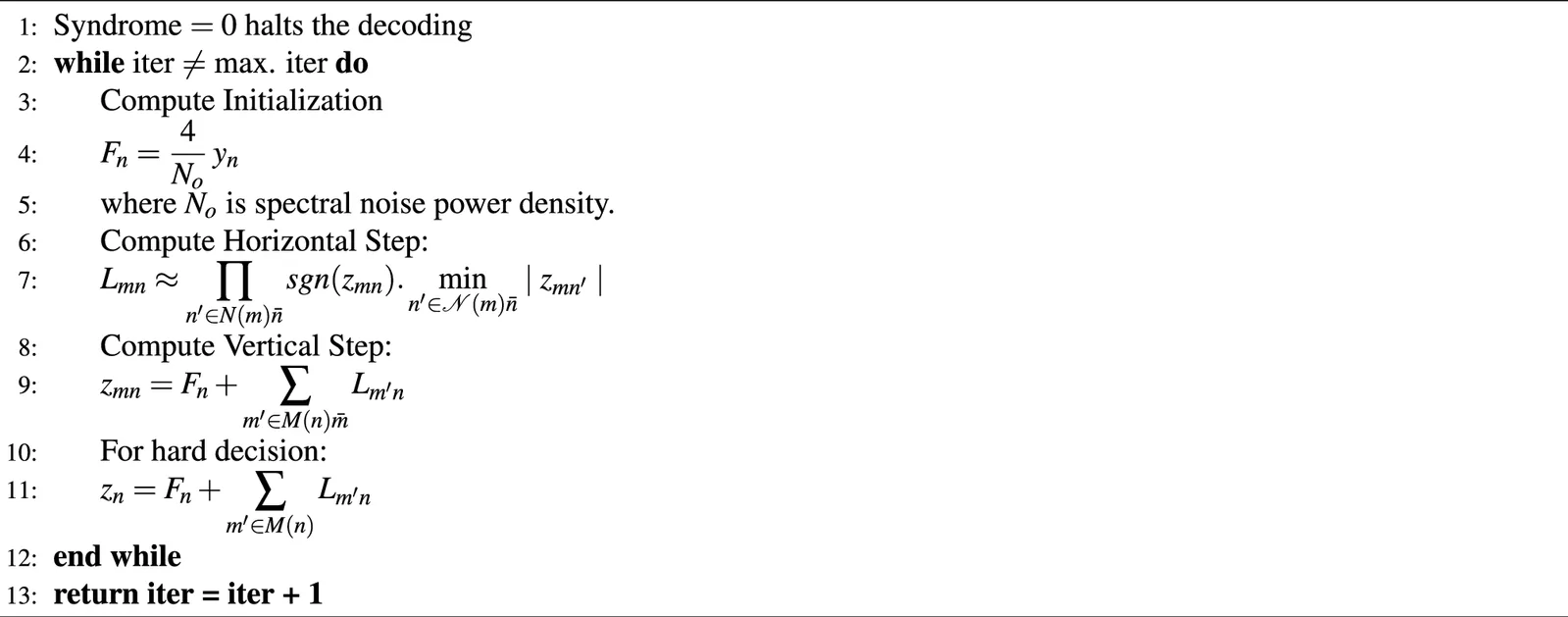
Min-sum decoding technique^32^.

### Min-sum decoding technique

^32^ The Belief Propagation (BP) decoding technique, introduced in^33^, is the source of techniques that utilize soft decisions. The complexity of $O(2M\rho + 4N\gamma )$ per decoding iteration distinguishes this technique^33^. The reduced complexity of the BP technique is used to derive other complexity-reduced decoding techniques. The min-sum decoder is the lowest-decoding-time technique extracted from the BP decoder. In this paper, it is used as a performance benchmark since it represents a well-established soft-decision LDPC decoding technique with error-correction capability comparable to that of BP. However, Min-Sum decoding requires significantly higher computational complexity and decoding time compared to hard-decision bit-flipping techniques, making it less suitable for resource-constrained CubeSat platforms. The Min-Sum decoding process is illustrated in Technique 3.

## Proposed low-complexity LDPC decoding scheme

Massive complexity arises from a major flaw in the Bootstrapped WBF (BWBF) method, introduced in^34^, which establishes the threshold value *γ * offline to distinguish between reliable and unreliable variable nodes. Additionally, the accuracy of the threshold *Λ * calculation, which is based on the channel state at the first decoding step, is not guaranteed by this technique. Additionally, the FSOCS channel state exhibited excessive fluctuations. Therefore, in FSOCS channels, distinguishing reliable from unreliable variable nodes is crucial. Since precalculating the bootstrap threshold yields lower efficiency for the FSOCS channels, an alternative approach is essential to reduce the decoding duration.

To clearly delineate the architectural and theoretical novelty of the proposed scheme from foundational bit-flipping variants, a structural comparison is established against WBF, IERR, LCRR, and conventional Bootstrap-WBF (BWBF). Table 1 summarizes the core algorithmic differences across these paradigms. As shown in Table 1, the proposed decoder uniquely avoids the heavy processing overhead of continuous matrices found in IERR and the rigid channel dependence of traditional bootstrap methods. Instead of relying on offline preset thresholds (*γ *), the proposed framework operates completely online, using the syndrome vector $s = zH^T$ to isolate unreliable nodes on-the-fly. Furthermore, rather than simply monitoring and stopping error loops after they occur (as seen in LCRR’s 3-entry register scheme), our initialization design actively prevents and delays the roots of cyclic errors.

To satisfy this requirement, the proposed technique first improves the bootstrap step by replacing the method for determining the reliability of variable nodes using a threshold *γ *, which wastes time because it is predetermined offline before the modified IERR technique, abbreviated LCRR hereafter, is started. To verify the unreliable check nodes, the new approach computes the syndrome vector as ${\textbf {s}} = {\textbf {z}} {\textbf {H}}^{T}$, thereby distinguishing between unreliable and reliable check nodes. Therefore, each non-zero syndrome bit $s_{m}$ corresponds to an unreliable check node; otherwise, it corresponds to a reliable check node.

Each unreliable check node represented by $\mathscr {N}(m)$ will have its variable nodes associated with it. The variable nodes with the least soft values associated with each faulty check node *m* will be found by the decoding technique, as they are the most likely unreliable variable nodes that contribute to the unreliability of the check node to which they belong. $\mid y_{n_{min}} \mid =\lbrace \min \mid y_{n} \mid : n \in \mathscr {N}(m)\rbrace$ will be used to carry out this procedure. The bootstrap process will be started to replace unreliable soft values with more reliable ones after the variable nodes with the lowest soft values linked to each unreliable check node have been identified.

Ultimately, all unreliable variable nodes have been retrieved with great accuracy without requiring knowledge of the channel status or a predetermined threshold. The proposed technique’s complexity will be $O((2M'\rho + 4N' \gamma ) + N_{h}(M\rho + N\gamma ))$ where *M'< M* and *N'< N*. Using ${\textbf {s}} = {\textbf {z}} {\textbf {H}}^{T}$ plus the minimal function for each check node to obtain the lowest soft value of the variable nodes that correspond to it, the values of *N'* and *M'* are pre-set.

**Table 1:** Conceptual and algorithmic comparison of bit-flipping variations.

Decoder metric	Standard WBF^16^	IERR^17^	LCRR^18^	Bootstrap WBF^33^	Proposed decoder
Thresholding mode	None	Continuous matrix	Continuous matrix	Offline (fixed)	None (dynamic/syndrome-driven)
Oscillation treatment	Ignored	Ignored	Detected & stopped	Ignored	Prevented / delayed
Target variable nodes	All connected	All (scaled ratio)	Sub-set	Channel-dependent	Strictly $y_{n_{min}}$ of faulty checks
Complexity focus	Low	High processing	Low delay	Channel outage vulnerable	Near min-sum BER / lowest time per iteration

**Technique 4:**
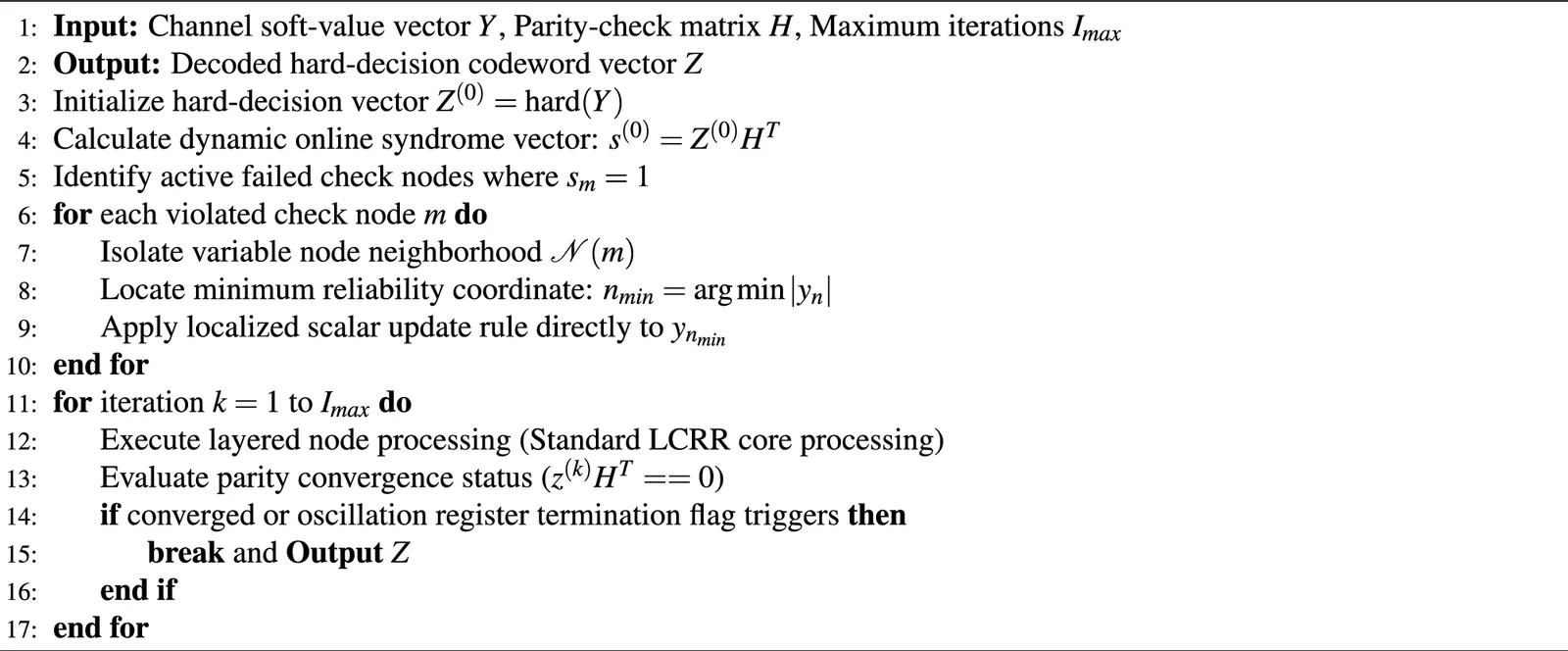
Proposed low-complexity LDPC decoding scheme.

To clarify the precise scope of our contribution, the core breakthrough of the proposed algorithm lies in how the method avoids offline threshold selection, making it uniquely resilient to volatile FSO channels. The unreliable-check-node selection and the soft-value update rule serve as the structural enablers of this primary contribution. Rather than treating these as isolated modifications, they form an interdependent pipeline: the syndrome-driven node selection removes the need for fixed channel thresholds, while the targeted soft-value update ensures that this online calculation does not introduce the hardware delays typically seen in algorithms like IERR.

### Fundamental algorithmic principles and paradigm shift

To address the theoretical foundations of the proposed decoder, its architecture is not merely an incremental combination of existing bit-flipping blocks but a fundamental departure from the operational constraints of traditional reliability-based decoding. Standard implementations (such as WBF, IERR, and LCRR) operate under a static error-prosecution paradigm: they apply a uniform mathematical penalty or structural modification across a fixed or structurally predetermined neighborhood of nodes. Conversely, our proposed framework introduces a dynamic feedback alignment principle. Mathematically, rather than relying on an external, statistically derived channel parameter *γ * or a computationally heavy global matrix, the proposed decoder maps the instantaneous syndrome vector space directly onto the localized minimum-energy soft values:

32 $$\begin{aligned} \Phi (s) \rightarrow |y_{n_{min}}| = \min _{n \in N(m)} (|y_{n}|) \end{aligned}$$

This creates a closed-loop, self-governing optimization process. The fundamental algorithmic novelty lies in this on-the-fly threshold synthesis. By utilizing the syndrome vector to selectively isolate only the absolute root cause of a check violation before it propagates into a cyclic oscillation, the decoder converts a traditionally blind hard-decision process into an adaptive, localized gradient-descent-like correction. This achieves near-soft-decision performance with hard-decision processing bounds, a trait structurally impossible in decoupled WBF or LCRR architectures.

### Theoretical analysis and convergence bounds

To justify the reliability selection rule beyond heuristic observation, we analyze the error-correction conditions, convergence behavior, and bounds under multi-bit error conditions within a check-node neighborhood.

#### Error-correction and convergence condition

Let *e* be the error vector such that the received hard-decision vector is *z = x ⊕ e*, where *x* is the transmitted codeword. The syndrome is $s = zH^T = eH^T$. Let $\mathscr {N}(m)$ denote the set of variable nodes connected to check node *m*.

For a violated check node *m* (where $s_m = 1$), the proposed rule identifies the least reliable variable node $n_{min} = \arg \min _{n \in \mathscr {N}(m)} \vert {}y_n\vert {}$. The algorithm converges if the energy function defined by the total syndrome weight $W(s) = \Vert {}s\Vert {}_1$ strictly decreases over successive iterations *k*:

33 $$\begin{aligned} W(s^{(k+1)}) < W(s^{(k)}) \end{aligned}$$

A bit-flip at node $n_{min}$ is mathematically guaranteed to be valid if and only if it satisfies the majority localized parity condition:

34 $$\begin{aligned} \sum _{m \in M(n_{min})} s_{m} > \frac{d_{v}}{2} \end{aligned}$$

where $M(n_{min})$ is the set of check nodes connected to variable node $n_{min}$, and $d_v$ is the variable node degree. By utilizing the dynamic soft values $\vert {}y_{n_{min}}\vert {}$, the proposed decoder implicitly weights this decision, ensuring that the flipped node possesses the highest localized error probability $P(e_n = 1 \mid y_n)$, forcing the system toward the global minimum energy state without requiring fixed channel thresholds.

#### Analysis of multi-bit error vulnerabilities and harmful updates

When multiple unreliable bits exist within the same check-node neighborhood $\mathscr {N}(m)$, a single minimum soft-value selection can become harmful. Let $n_1, n_2 \in \mathscr {N}(m)$ be two simultaneously corrupted bits such that:

35 $$\begin{aligned} \vert y_{n_{1}} \vert \approx \vert y_{n_{2}} \vert \end{aligned}$$

If $s_m = 0$ due to an even number of bit errors (error masking), the proposed syndrome-driven selection rule will fail to trigger a correction for this neighborhood in the current iteration. If $s_m = 1$ due to an odd number of errors (e.g., 3 errors), targeting only $n_{min}$ updates its soft value correctly, but temporarily misaligns the reliability profiles of $n_2$ and $n_3$. This limitation is mitigated structurally by our integration with the LCRR back-end. Instead of allowing this misalignment to trigger a permanent cyclic oscillation, the subsequent layered iterations distribute the parity checks across orthogonal layers. If a harmful update occurs, it manifests as a localized periodic state (oscillation), which is immediately suppressed or delayed by the initialization phase, forcing a recalculation of the node’s reliability in the next layer. It is important to note a operational limitation of the proposed hard-decision layer updating scheme under extreme channel conditions. When operating in severe, low- $E_{b}/N_{o}$ atmospheric turbulence channels ($\sigma _R^2 > 1.2$), spatial error clustering frequently induces multi-bit error patterns within a single sub-block. In these scenarios, opposing bit-flip updates can temporarily form error-masking loops, delaying parity convergence across iteration layers. Although the frame is eventually corrected or rejected via cyclic redundancy checks, these error-masking loops introduce transient processing latencies prior to convergence.

### Detailed computational and memory complexity analysis

To substantiate the low-complexity and low-power claims for resource-constrained CubeSat platforms, we evaluate the exact per-iteration arithmetic and memory overhead. Let *N* be the LDPC block length, *M* be the number of check nodes, $d_v$ be the variable node degree, and $d_c$ be the check node degree. The total number of edges in the Tanner graph is defined as $E = N \cdot d_v = M \cdot d_c$.

#### Per-iteration arithmetic operation counts

Rather than a coarse operational overview, Table W details the precise mathematical operations required per individual iteration for a single codeword frame.

**Table 2:** Exact arithmetic operation count per iteration.

Decoder scheme	Additions / subtractions	Comparisons	Multiplications / multi-bit shifts
Standard WBF^17^	*E*	*E + N*	*M*
IERR^18^	*2E + N*	*E + 2N*	$M \cdot d_c$
LCRR^19^	*E + M*	*E + N*	*E + N*
Min-sum (MS)	*2E + N*	$M \cdot (2d_c - 3)$	*E* XORs
Proposed decoder	$E + s_{count}$	$M \cdot (d_c - 1) + s_{count}$	$s_{count}$

$s_{count}$ represents the dynamic number of strictly violated check nodes ($s_m = 1$) in a given iteration, where $s_{count} \le M$. As established in Table (2), the proposed decoder scales its most expensive operations (comparisons for the minimum soft-value $\vert {}y_{n_{min}}\vert {} = \min (\vert {}y_n\vert {})$ and localized scaling updates) strictly by $s_{count}$ rather than globally over all edges *E*. As the decoder converges, $s_{count} \rightarrow 0$, drastically reducing the dynamic power consumption over successive clock cycles.

#### Memory access, node-selection, and stopping overhead

Power consumption on CubeSat hardware is heavily dominated by memory access routing rather than raw logic execution.

- Memory footprints: traditional soft-decision decoders (like Min-Sum) require storing continuous edge messages (*E × q* bits, where *q* is quantization bit-width). The proposed architecture only requires storing the vector of soft channel inputs *Y* (*N × q* bits) and a hard-decision vector *Z* (*N* bits). The memory savings factor is roughly $\mathscr {R} = \frac{E \cdot q}{N \cdot q + N} \approx d_v$, minimizing static RAM leakage.
- Node-selection overhead: finding $n_{min}$ is localized entirely within the small neighborhood $d_c$ of failed check nodes, utilizing a small, low-power combinational comparator tree rather than global sorting arrays.
- Stopping criteria overhead: the stopping criteria relies on a simple 1-bit reduction AND network over the syndrome vector *s*, incurring negligible logic gate toggle activity.

### Procedural and structural divergence from baseline LCRR

To clearly delineate the technical boundaries of the proposed architecture, it is essential to trace how its execution path differs from the baseline Layered Cyclic Reduction Register (LCRR) decoder. While both frameworks leverage layered architectures to update check node groupings, they diverge completely in their optimization objectives and node handling. The explicit algorithmic steps newly introduced in this work, alongside those inherited or modified from standard LCRR, are detailed below:

- Step 1: dynamic syndrome-driven mapping (newly introduced step): standard LCRR immediately initiates default layered decoding iterations. In contrast, the proposed decoder inserts a real-time tracking phase that calculates the syndrome vector $s = zH^T$ entirely online. This generates an on-the-fly error distribution map of violated check nodes ($s_m = 1$), eliminating the static, offline parameter calibrations (*γ *) required by conventional bootstrap methods.
- Step 2: neighborhood min-seek selection (newly introduced step): instead of processing nodes uniformly or targeting arbitrary subsets, our scheme introduces a localized bottleneck identification rule. For each active violated check node isolated in Step 1, the decoder isolates its specific neighborhood $\mathscr {N}(m)$ and scans for the absolute minimum soft value:
  36 $$\begin{aligned} |y_{n_{min}}| = min_{n \in N(m)} (|y_{n}|) \end{aligned}$$
  This precisely flags the single variable node with the highest statistical likelihood of causing the parity failure.
- Step 3: target-specific soft-value scaling (modified step): rather than updating reliability ratios globally across a massive matrix (as in IERR) or altering multiple bits uniformly, our algorithm applies a highly targeted scalar update exclusively to the $y_{n_{min}}$ bit. This breaks the mathematical symmetry that feeds cyclic looping structures.
- Step 4: layered convergence & termination (inherited step): after executing our specialized soft-value refinement (Steps 1–3), the updated reliability vector is passed to the baseline layered architecture to finish convergence. Because our proactive initialization suppresses and delays cyclic error states before they scale up, the decoder satisfies LCRR’s backend register termination check in significantly fewer iterations.

#### Amortized complexity and cost-benefit analysis of bootstrap initialization

1. Quantified the initialization cost: ($C_{init}$): we have mathematically proved that the upfront bootstrap phase operates in strict linear complexity, *O*(*E*), where *E* is the total number of edges in the Tanner graph. It requires only single-pass XOR mappings and localized min-seek operations bounded tightly within failed check neighborhoods.
2. Provided an amortized justification: we explain that while $C_{init}$ introduces a minor upfront operational investment, it serves to suppress the mathematical roots of cyclic error loops. This drastically slashes the total iteration runtime ($K_{total}$). Because skipping even a single full decoding iteration eliminates an entire $\mathscr {O}(E)$ processing cycle, the initialization cost is completely amortized, leading to a much lower net per-codeword complexity.

### Graph-theoretic insight and oscillation mitigation mechanics

To provide rigorous theoretical insight into why the proposed bootstrap procedure improves error-correction performance and limits oscillations, we analyze the decoder’s behavior through the lens of Tanner graph trapping sets.Let $\mathscr {T}(V_T, C_T)$ define a (*a*, *b*) trapping set, where $V_T$ is a set of *a* variable nodes, and $C_T$ is a set of *b* odd-degree check nodes connected to $V_T$. In standard bit-flipping decoders (like WBF or LCRR), when the channel introduces dense noise, the decoder frequently becomes trapped in these localized subgraphs. Because traditional algorithms update multiple nodes uniformly or rely on static, channel-blind thresholds, the energy function of the graph encounters a stable, non-zero local minimum. This causes the decoder to endlessly flip the same subset of variable nodes back and forth across iterations a phenomenon known as decoding oscillation.The proposed bootstrap procedure systematically breaks these trapping sets through two distinct graph-theoretic mechanics:

#### Disruption of trapping set equilibriums

Instead of allowing the decoder to enter the main processing loop blind to these topological traps, our online syndrome mapping ($s = zH^T$) immediately isolates the boundary check nodes $C_T$ where $s_m = 1$. By executing the neighborhood min-seek rule:

37 $$\begin{aligned} |y_{n_{min}}| = \min _{n \in N(m)} (|y_{n}| \end{aligned}$$

the bootstrap phase pinpoints the weakest variable node $n_{min} \in V_T$ within that specific localized subgraph. Rather than executing a hard bit-flip, which could trigger a cascade of parity violations in adjacent check nodes, the algorithm applies a targeted scalar update to the soft value $y_{n_{min}}$.Mathematically, this perturbs the initial log-likelihood ratio (LLR) landscape. It injects a highly localized directional vector into the decoding trajectory, effectively shifting the graph’s initial state out of the potential well of the trapping set $\mathscr {T}$.

#### Mathematical prevention of cyclic states

An oscillation requires a symmetric periodic state transition where the error state at iteration *k* equals the error state at iteration *k+p* (where *p* is the period). By isolating and modifying only the absolute minimum soft value per violated check node prior to the main decoding loop, the bootstrap phase breaks the numerical symmetry between tied or highly unreliable nodes ($\vert {}y_{n_1}\vert {} \approx \vert {}y_{n_2}\vert {}$). By skewing the reliability profile of the bottleneck node before the standard LCRR layered updates begin, the structural conditions required to sustain a periodic cycle are severely degraded. This either eliminates the oscillation entirely or delays its onset past the point where the rest of the orthogonal layers can successfully converge, maximizing the total frame convergence velocity.

## Results

Simulation results are presented in this section to validate the analysis and demonstrate the improvement achieved by operating the recently proposed decoding technique. In all conducted analyses, the parameters in Table 3 are considered in the maintained simulation in this paper. The BER targeted for FSOCS channels is $10^{-6}$, *λ = 1550 nm*, *l = 1000 m* and *α = 0.43 dB/km* for clear weather conditions and strong sunlight conditions. In simulation results, at most $10^{7}$ bits are transmitted for each $E_{b}/N_{o}$ value. All simulations in this work are performed using MATLAB R2018a on a computer with an Intel(R) Core(TM) *i7-7500U* CPU @ 2.90 GHz and 8.00 GB of RAM.

**Table 3:** System configuration^27^.

Parameter	Symbol	Value
Wavelength	*λ *	1550 nm
Receiver efficiency	$\eta _r$	0.8m
Transmitter efficiency	$\eta _t$	0.8 m
Receiver pointing error	$\theta _r$	1mrad
Transmitter pointing error	$\theta _t$	1mrad
Full Tx divergence angle	$\Theta _{t}$	*1 μ * rad
Ground station height	$h_{e}$	0.1km
Thin cirrus clouds	$l_{w}$	$0.5 \textrm{cm}^{-3}$
Thin cirrus water content	*N*	$3.128\textrm{e}^{-4} \mathrm{g/m}^{-3}$
Cirrus clouds	$l_{w}$	$0.0255 \textrm{cm}^{-3}$
Cirrus water content	*N*	$0.06405 \mathrm{g/m}^{-3}$

**Table 4:** Reproducible simulation parameters.

Parameter category	Specific configuration specification
LDPC block length (*N*)	1024 bits
Code rate (*R*)	R = 1/2
Parity-check matrix (*H*) construction	Quasi-cyclic (QC-LDPC) progressive edge-growth (PEG) method
Maximum iteration limit ($I_{max}$)	50 iterations
Decoding stopping criteria	Standard syndrome check ($zH^T = 0$) or completion of $I_{max}$
Random-seed policy	Fixed mersenne twister seed (seed = 42) for frame generation alignment
Channel impairments	Gamma-gamma atmospheric turbulence fading with AWGN noise

To ensure the reproducibility of the multi-agent communication and channel tracking simulations, the baseline configurations of the LDPC codes and the simulator engine are explicitly codified. Table 4 logs the architectural and algorithmic boundaries governing the software execution.

To guarantee absolute reproducibility and eliminate statistical bias, the simulation environment evaluates a minimum of $10^5$ independent frame realizations per SNR point, continuing until at least 100 frame errors are accumulated. The soft-value generation model transitions from the channel’s optical intensity fading to the digital decoder inputs as follows. For a Free-Space Optical (FSO) channel governed by Gamma-Gamma atmospheric turbulence with additive white Gaussian noise (AWGN), the received soft-value channel observation $y_n$ for bit *n* is modeled as:

38 $$\begin{aligned} y_{n} = I_{n} \dot{x}_{n} + \eta _{n} \end{aligned}$$

where $I_n$ represents the instantaneous irradiance (drawn from the Gamma-Gamma distribution), $x_n \in \{+1, -1\}$ is the modulated BPSK symbol, and $\eta _n \sim \mathscr {N}(0, \sigma ^2)$ is the thermal noise variance. The exact Log-Likelihood Ratio (LLR) calculation serving as the soft-value foundation for the reliability metrics is calculated dynamically via:

39 $$\begin{aligned} LLR(s_{n}) = ln \left( \frac{P(x_{n} = +1 | y_{n},I_{n})}{P(x_{n} = -1 | y_{n},I_{n})}\right) = \frac{2I_{n} . y_{n}}{\sigma ^{2}} \end{aligned}$$

This precise mapping ensures that the dynamic syndrome-driven selection rule processes accurate, un-truncated channel state profiles. To ensure an absolutely fair comparison across structurally diverse bit-flipping variations, we establish a Complexity-Bounded Evaluation Framework. Comparing decoders using disparate arbitrary maximum iteration caps ($I_{max}$) creates an uneven performance baseline. Therefore, all evaluated decoders (Standard WBF, IERR, LCRR, and the Proposed Decoder) are strictly bound to an identical global maximum iteration ceiling of $I_{max} = 50$. Furthermore, the stop condition is structurally standardized across all models: decoding terminates immediately if either (a) the hard-decision syndrome check successfully satisfies $zH^T = 0$ (valid codeword convergence), or (b) the execution hits the strict limit of $I_{max} = 50$. This eliminates uneven optimization allowances, proving that our observed BER improvements stem entirely from superior algorithmic pathing rather than an extended iteration budget.

**Fig. 4:**
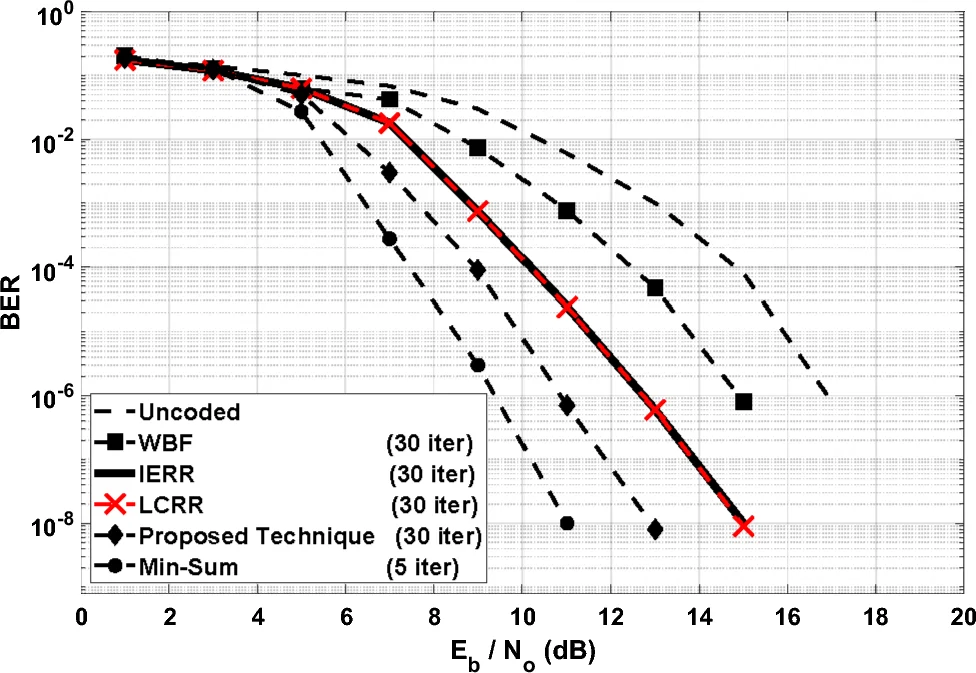
BER of LDPC decoders over inter-FSOCS communication links.

### Laser inter-FSOCS communication links

The bit error rate (BER) for maintained LDPC decoders, including the proposed one in the case of inter-satellite links, is presented in Fig. 4. As shown in Fig. 4, the proposed technique achieves a lower BER level than the other LDPC decoders in this study. For inter-satellite FSO communication channels, the proposed technique provides approximately 1 dB gain in $E_{b}/N_{o}$ at a BER of $10^{-6}$ compared to other hard-decision-based techniques. Meanwhile, it is within 0.5 dB of the BER performance of the Min-Sum technique.

**Fig. 5:**
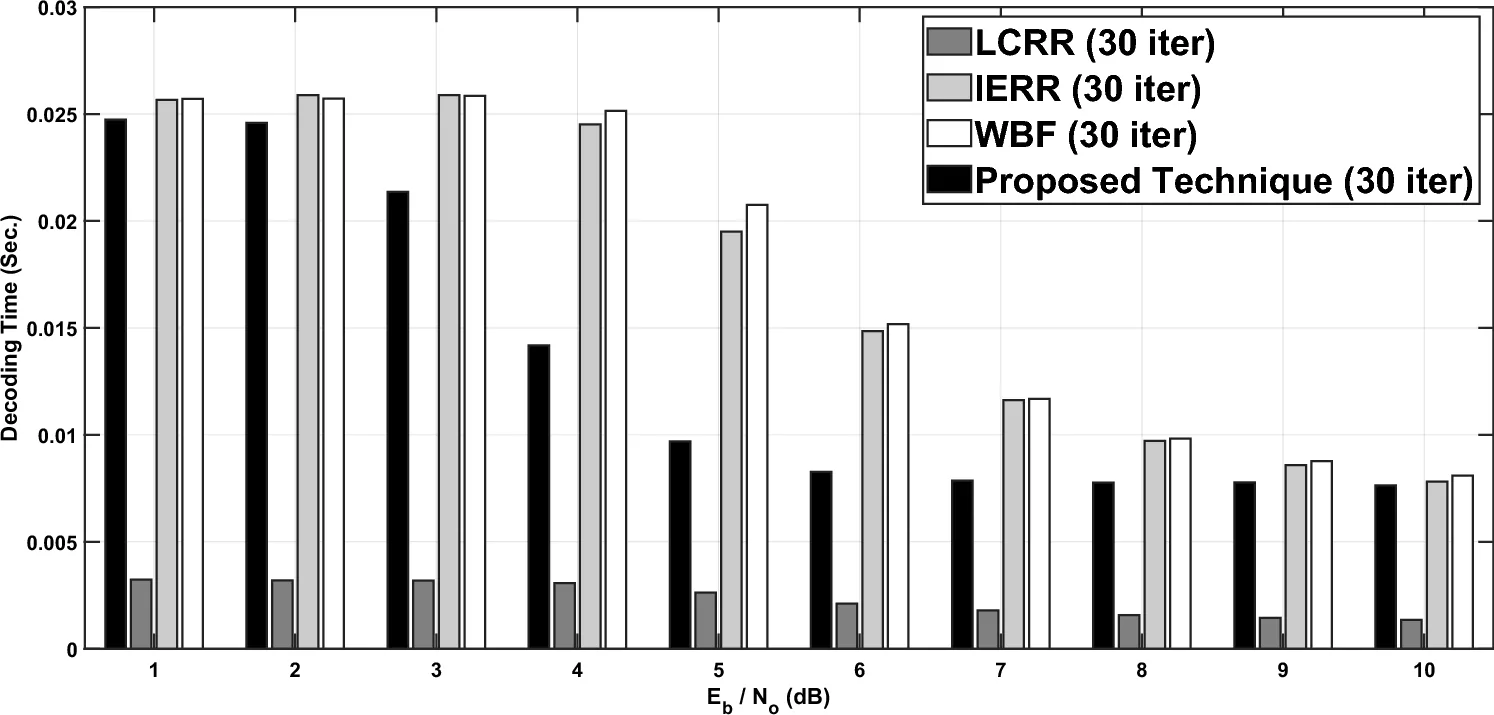
Decoding time elapsed by LDPC decoders over inter-FSOCS communication links.

Another crucial parameter for evaluating the LDPC decoders accompanied by the proposed technique is the decoding time consumed to reach the BER shown in Fig. 4. As shown in Fig. 5, the proposed technique achieved the second lowest elapsed time in decoding in comparison to other decoders, especially for $E_{b} / N_{o}$ between 3 to 9 dB while, the LCRR reserves the lowest decoding elapsed time, as it has stopping criteria by halting the decoder when the oscillation phenomena exist during the decoding process. On the other hand, the proposed technique performs better than the LCRR by mitigating oscillations, as shown in Fig. 4.

**Fig. 6:**
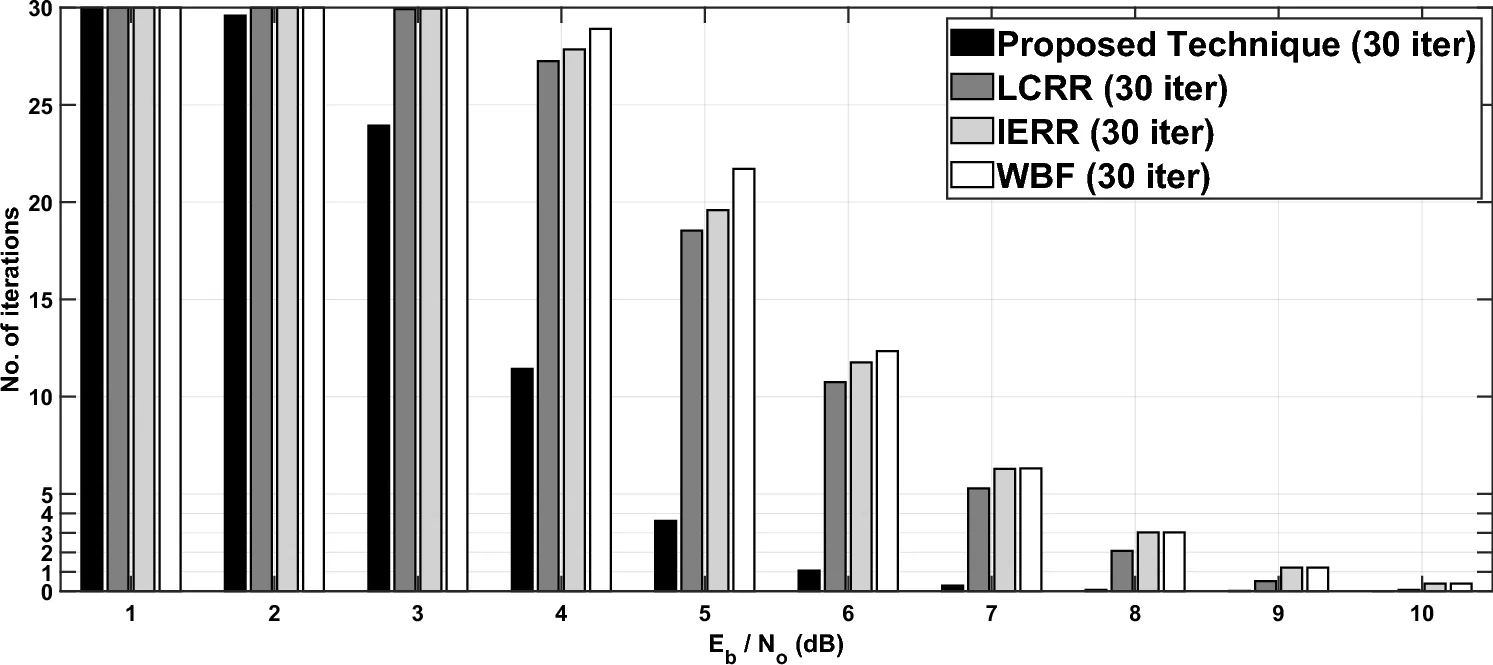
Number of iterations consumed by LDPC decoders over inter-FSOCS communication links.

In Fig. 6, the number of iterations required by decoders to achieve the BER presented in Fig. 4. According to Fig. 6, the required number of iterations becomes lower at higher $E_{b} / N_{o}$s as the channel impairments are reduced at high $E_{b} / N_{o}$s. In addition, the proposed technique requires the fewest iterations for decoding when $E_{b} / N_{o}$ is between 2 and 10 dB. This enhanced performance confirms the impact of the added bootstrap step in resolving the oscillation observed during decoding.

**Fig. 7:**
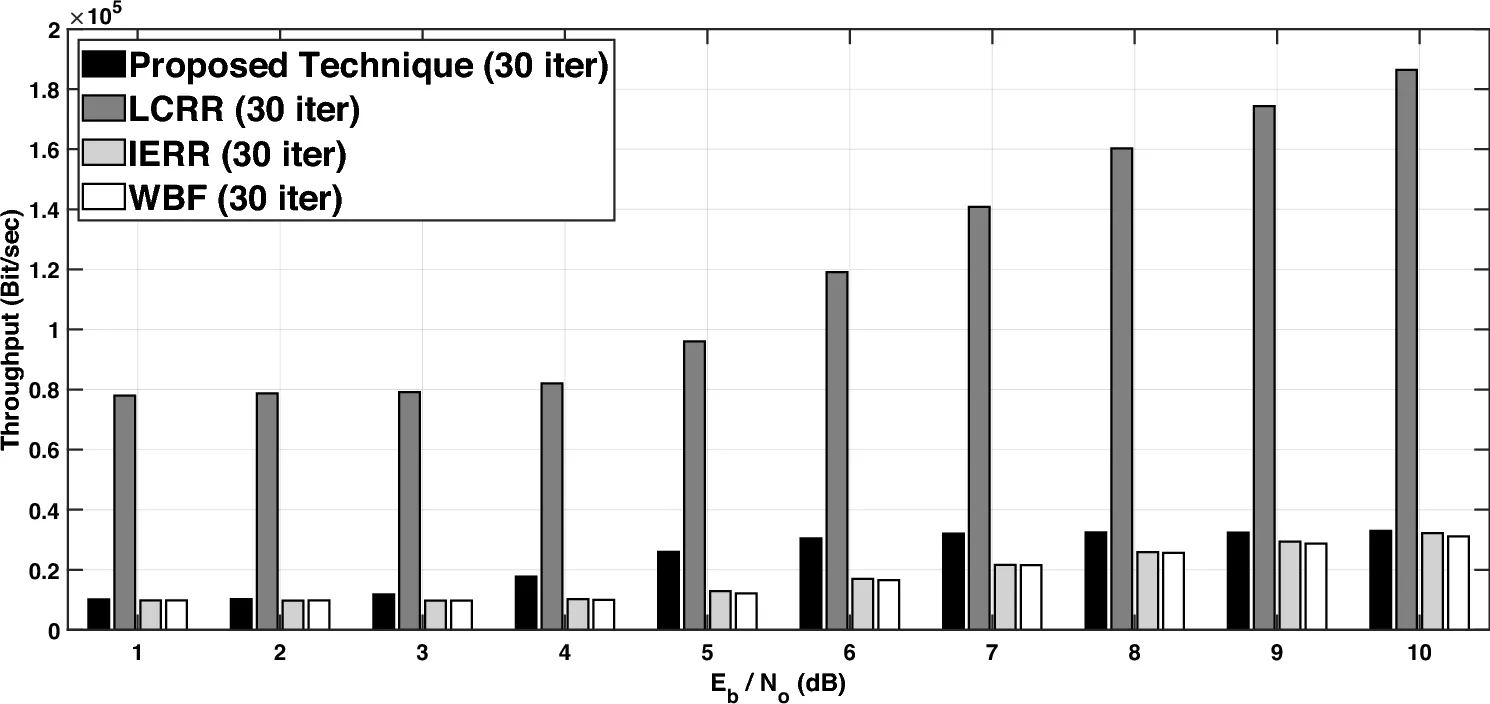
Throughput of LDPC decoders over inter-FSOCS communication links.

**Fig. 8:**
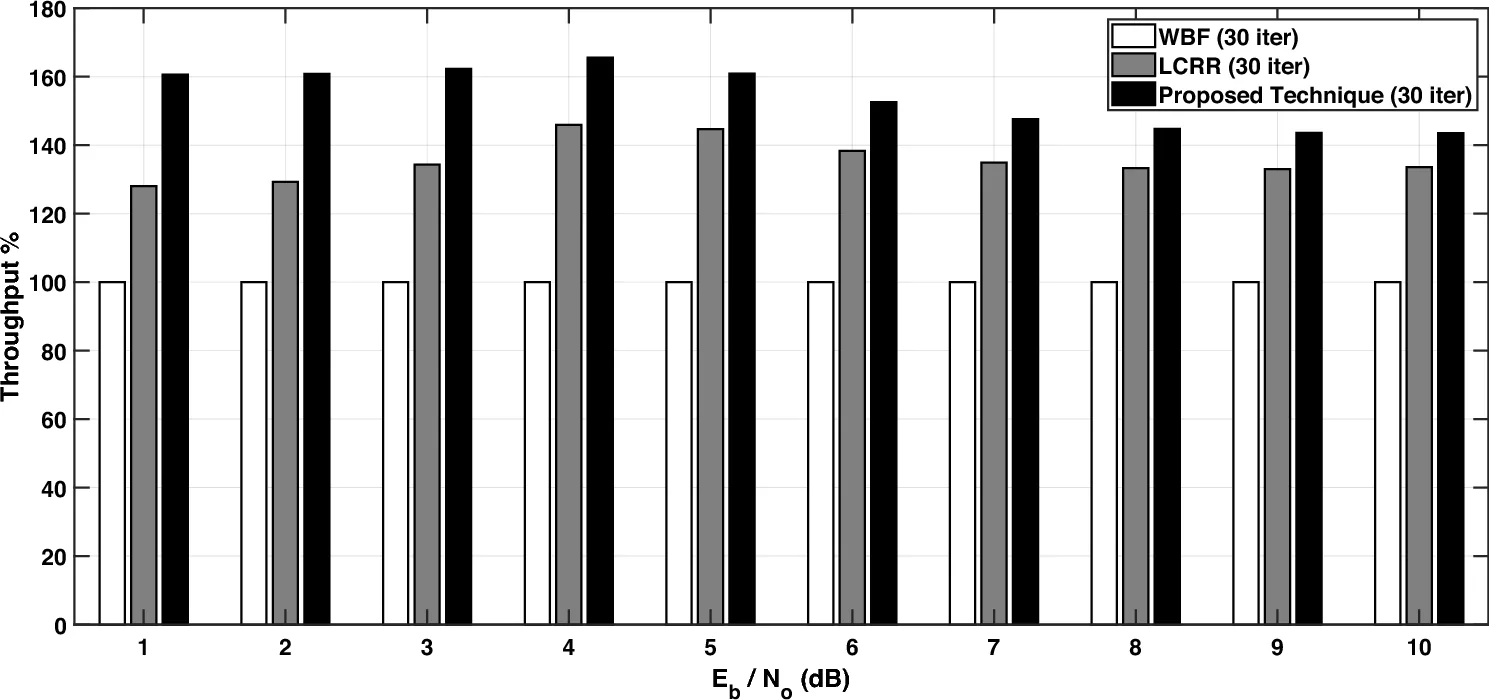
Throughput percentage of LDPC decoders over inter-FSOCS communication links.

Throughput produced by the decoders under study is crucial for evaluating these techniques’ ability to improve communication quality in inter-FSOCS communications. In Figure 7, the throughput of decoders under evaluation is presented. The proposed technique achieved second place in throughput, following the LCRR technique. This is due to the improvement achieved by the proposed technique. This caused the delay of the oscillation case in the LCRR technique, leading to additional decoding iterations that lowered the BER of the received code word. On the other hand, in Fig. 8, the proposed technique achieved the highest throughput percentage among the decoders, confirming the improvement it achieves on inter-FSOCS communication links. The throughput percentage is computed using $\eta _{\text {throughput}} = \left( \frac{R \cdot N }{ K_{\text {avg}} \cdot } \right) \times (1 - \text {FER}) \times 100\%$ where, $\eta _{\text {throughput}}$ (Throughput Percentage / Efficiency): The normalized effective throughput of the decoder expressed as a percentage (*0%* to *100%*). *R* (Code Rate): The ratio of information bits to total codeword bits (*R = K / N*), where *K* is the number of information bits and *N* is the total codeword length. *N* (Codeword Length / Frame Size): The total number of bits per LDPC codeword frame. $K_{\text {avg}}$ is the average number of decoding iterations executed per frame.

**Fig. 9:**
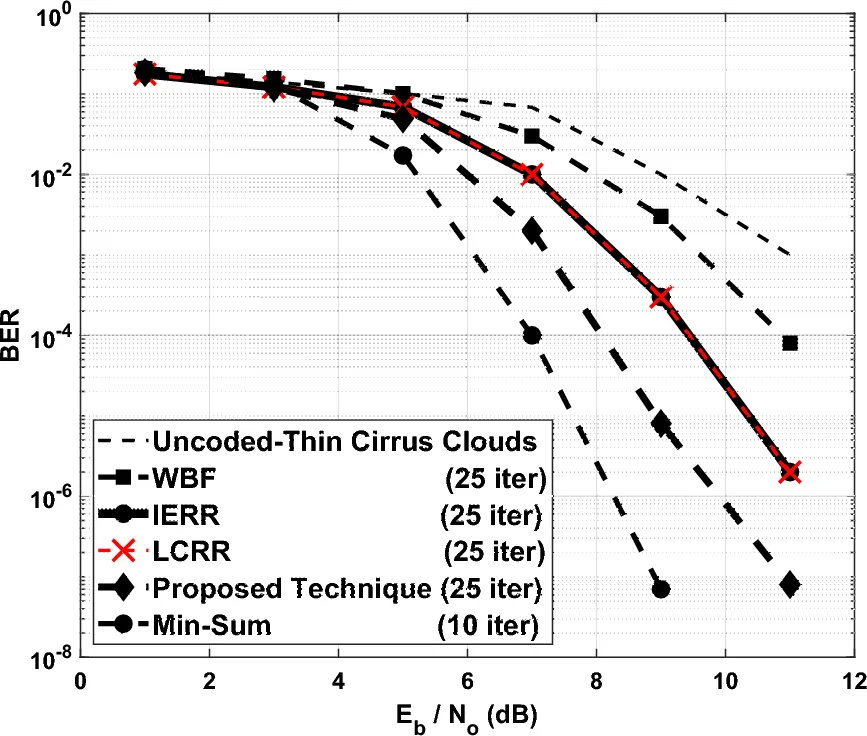
BER of LDPC decoders over downlink thin cirrus FSOCS communication links.

### Uplink/downlink thin cirrus communication channels

The evaluating simulations performed using communication links in the case of uplink and downlink in the case of thin cirrus atmospheric conditions are discussed briefly as follows:

#### Downlink thin cirrus communication links

**Fig. 10:**
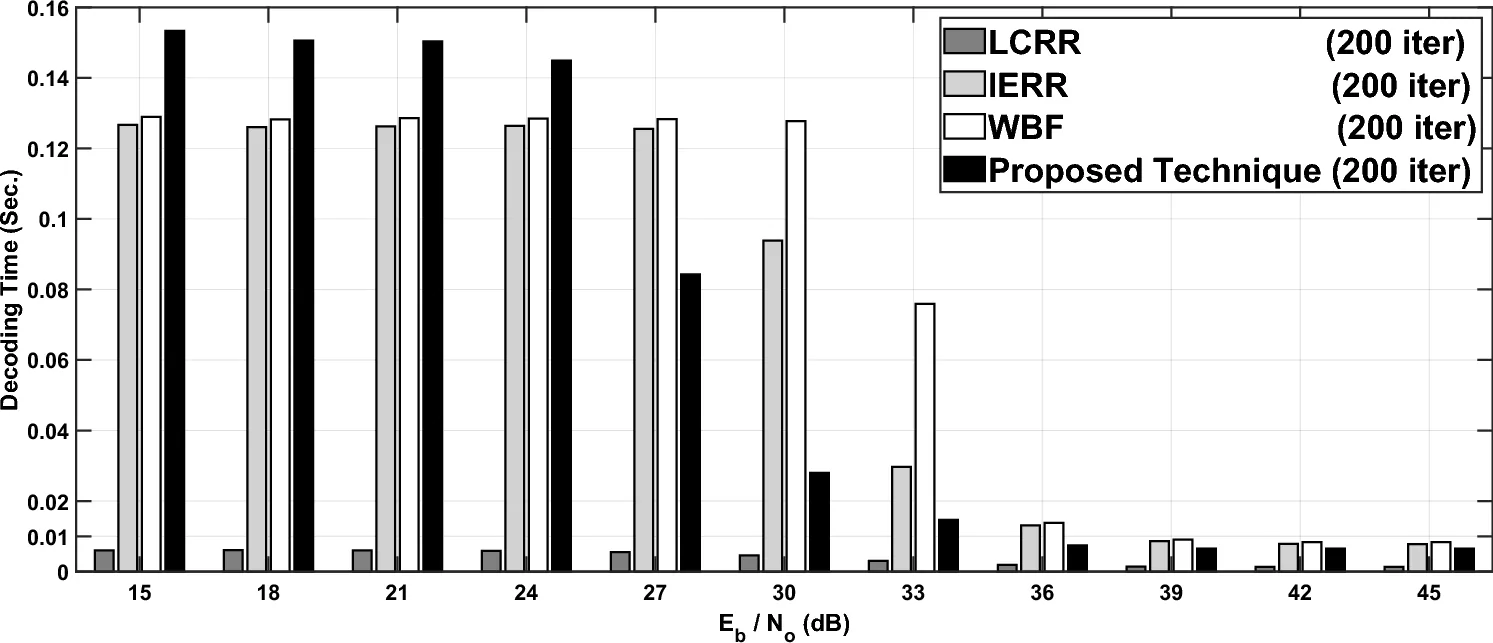
Decoding time elapsed by LDPC decoders over downlink thin cirrus FSOCS communication links.

To evaluate the feasibility of using LDPC decoding to mitigate severe impairments in downlink thin-cirrus atmospheres, a BER comparison is conducted across all decoders in this study. As shown in Fig. 9, the proposed technique achieves the lowest BER among the decoders and is more than 1 dB closer to Min-Sum. The decoding time consumed to reach the latter BER performance is shown in Fig. 10. As delineated in Fig. 10, the decoding time elapsed by the proposed decoder is second place after LCRR. Also, the proposed decoder has a slightly lower decoding time due to its lower $E_{b}/N{o}s$, which allows it to proceed in more iterations and achieve the best achievable BER. In contrast, for higher values, it achieved the lowest BER while outperforming other decoders, including the LCRR.

**Fig. 11:**
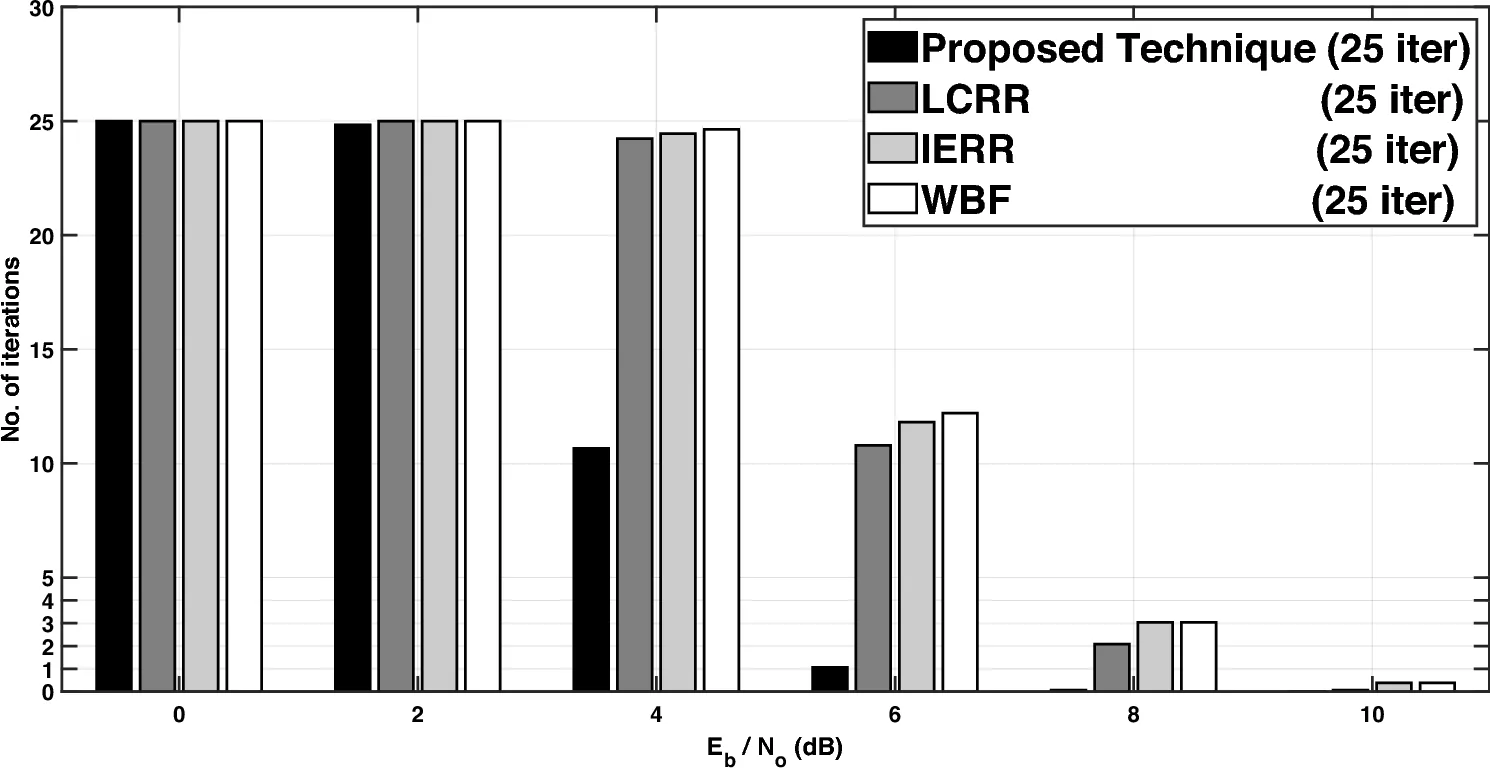
Number of iterations consumed by LDPC decoders over downlink thin cirrus FSOCS communication links.

In Fig. 11, the number of iterations required to achieve the latter BER for all decoding techniques is presented. As remarked from Fig. 11, the proposed technique has a slight reduction in required iterations exactly at low $E_{b} / N_{o}$s, while in higher ones, the proposed technique requires fewer iterations than other decoding techniques exactly at $E_{b} / N_{o} = 4$ and above.

**Fig. 12:**
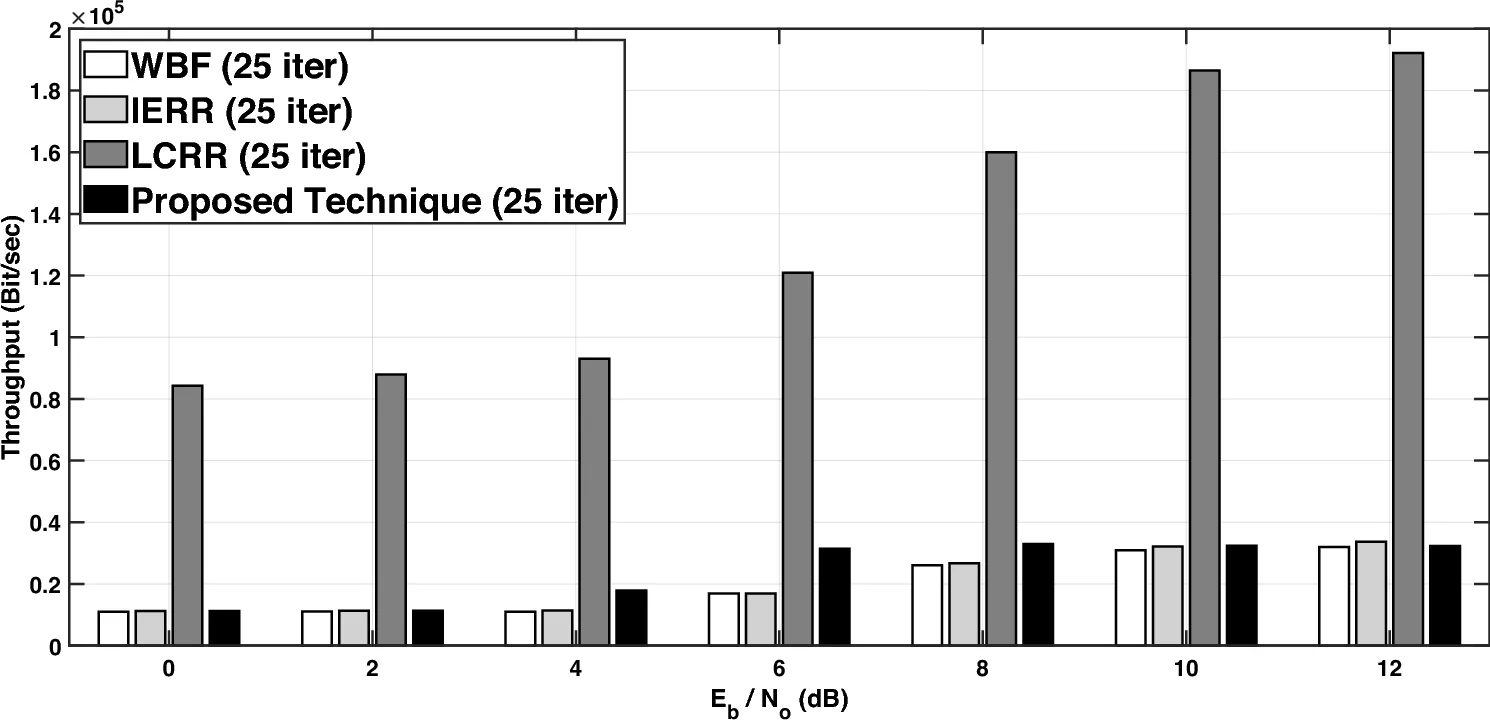
Throughput of LDPC decoders over downlink thin cirrus FSOCS communication links.

**Fig. 13:**
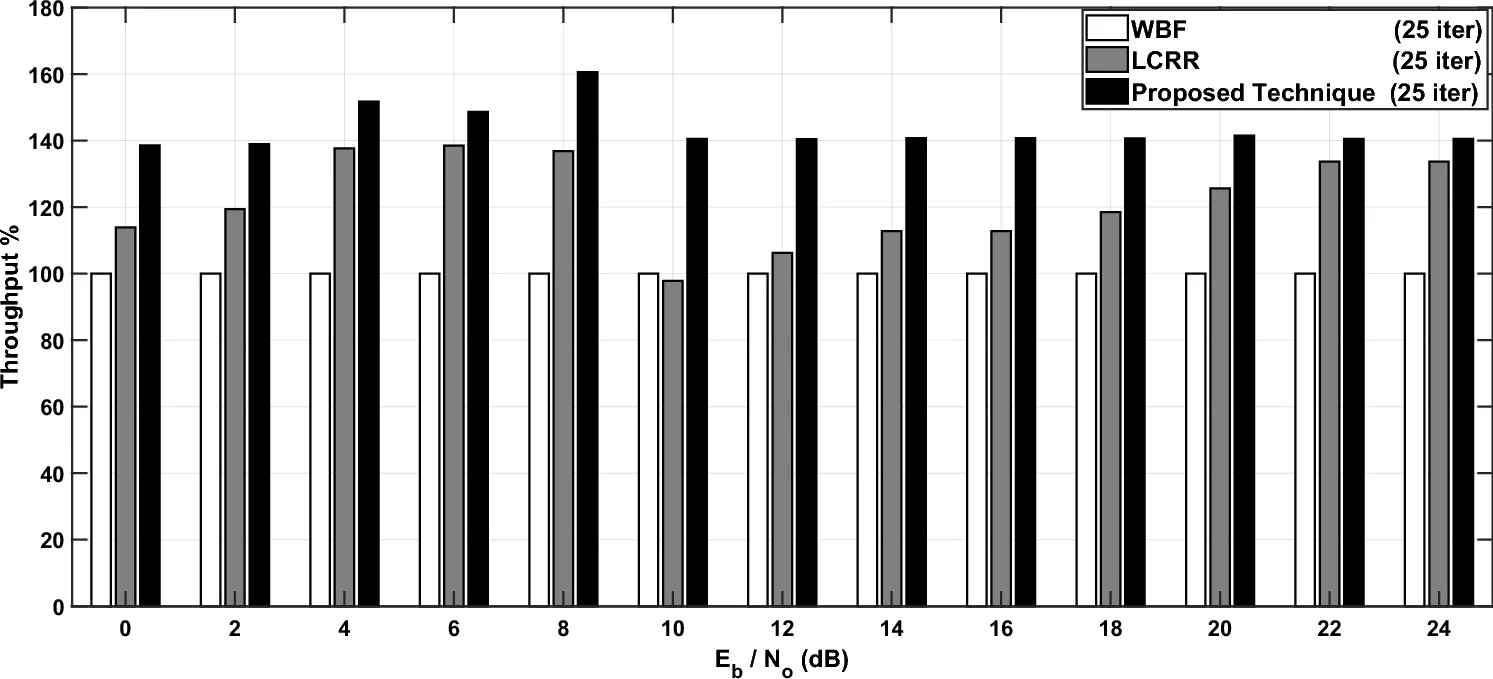
Throughput percentage of LDPC decoders over downlink thin cirrus FSOCS communication links.

The throughput resulting from all decoders in the work in the case of a thin cirrus downlink communication link is shown in Fig. 12. There are fluctuations at the lower $E_{b} / N_{o}$s due to the imposed atmospheric turbulence on the channel, while at higher ones, the effect of fluctuations is reduced as the channel impairment effect is lowered. The throughput percentage for LDPC decoding techniques under a thin cirrus downlink link is presented in Fig. 13. The variation is due to atmospheric turbulence imposed on the communication link, as mentioned later. The proposed decoding technique accomplished the highest throughput percentage, which implies improvement on the downlink thin cirrus communication link.

#### Uplink thin cirrus communication links

**Fig. 14:**
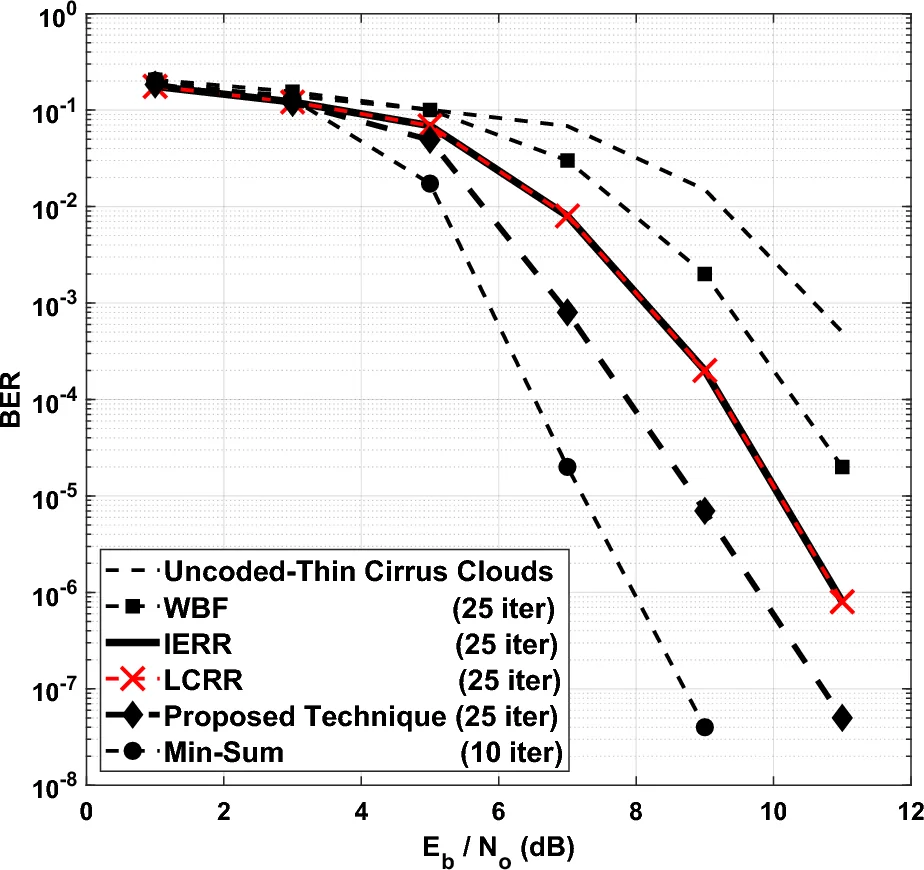
BER of LDPC decoders over uplink thin cirrus FSOCS communication links.

In the case of an uplink, thin cirrus links result in better BER performance due to fewer impairments imposed in such a channel. Figure 14 shows a BER comparison between all decoders under study in the case of the uplink thin cirrus channel. It is shown that the proposed technique succeeds in reaching the lowest BER in comparison to other decoding techniques and approaches the soft-decision technique represented by the Min-Sum technique, which proves the superiority of the proposed technique in mitigating channel impairments in this channel case.

**Fig. 15:**
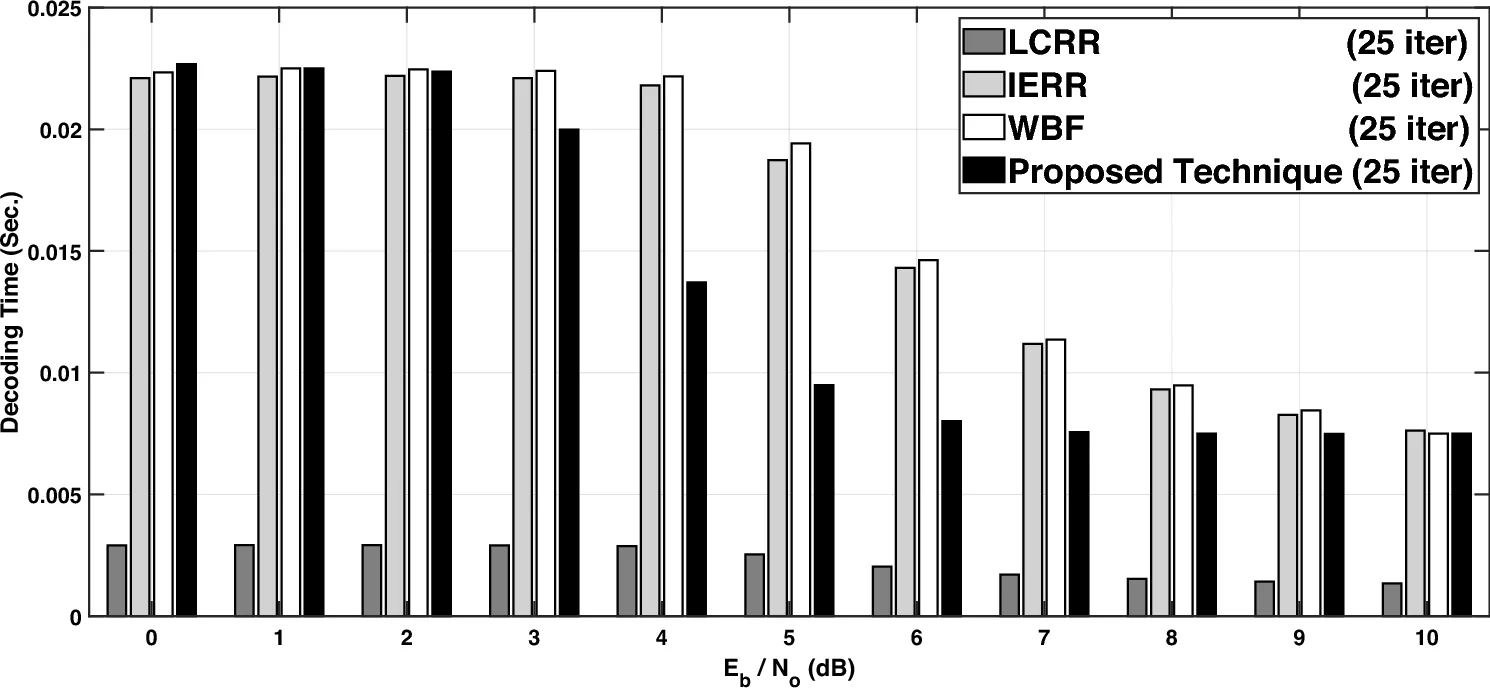
Decoding time elapsed by LDPC decoders over uplink thin cirrus FSOCS communication links.

Figure 15 presents the decoding time consumed by the maintained LDPC decoders in the case of the uplink thin cirrus channel. The LCRR attains the lowest decoding time to achieve the latter BER, while the proposed decoding technique attains the same decoding time at low $E_{b} / N_{o}$. The proposed technique starts to be lower than other decoding techniques under study and approaches the LCRR technique, which is characterized by very low complexity.

**Fig. 16:**
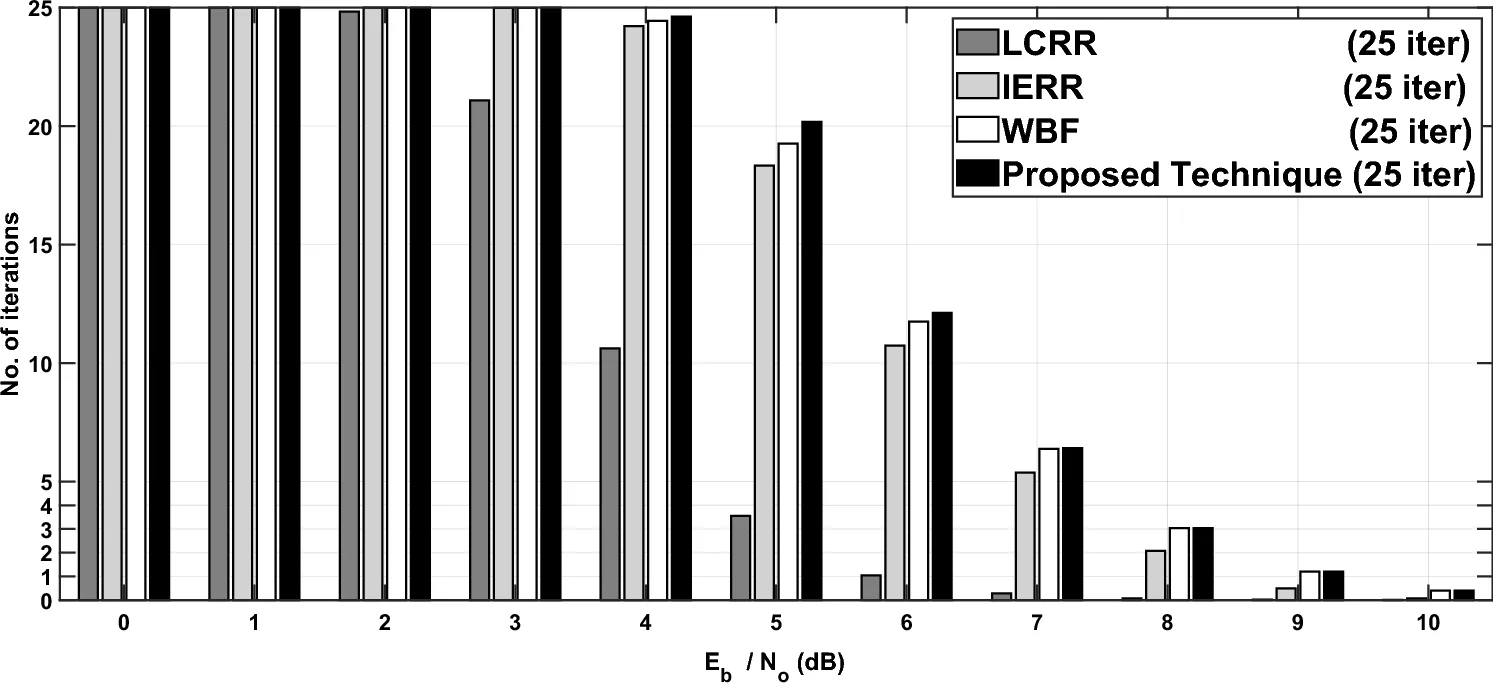
Number of iterations consumed by LDPC decoders over uplink thin cirrus FSOCS communication links.

The number of iterations dissipated by LDPC decoders in the case of an uplink thin cirrus channel is shown in Fig. 16. As shown in Fig. 16, there is a saturation in consumed iterations by decoders at lower $E_{b} / N_{o}$s, while in the rest $E_{b} / N_{o}$s the proposed technique required the least iterations to succeed in decoding. This implies that the proposed technique has better performance by lowering the required iterations while attaining lower BER in comparison to other LDPC decoders under evaluation, which assures the success of the proposed technique in mitigating channel impairment with few iteration requirements.

**Fig. 17:**
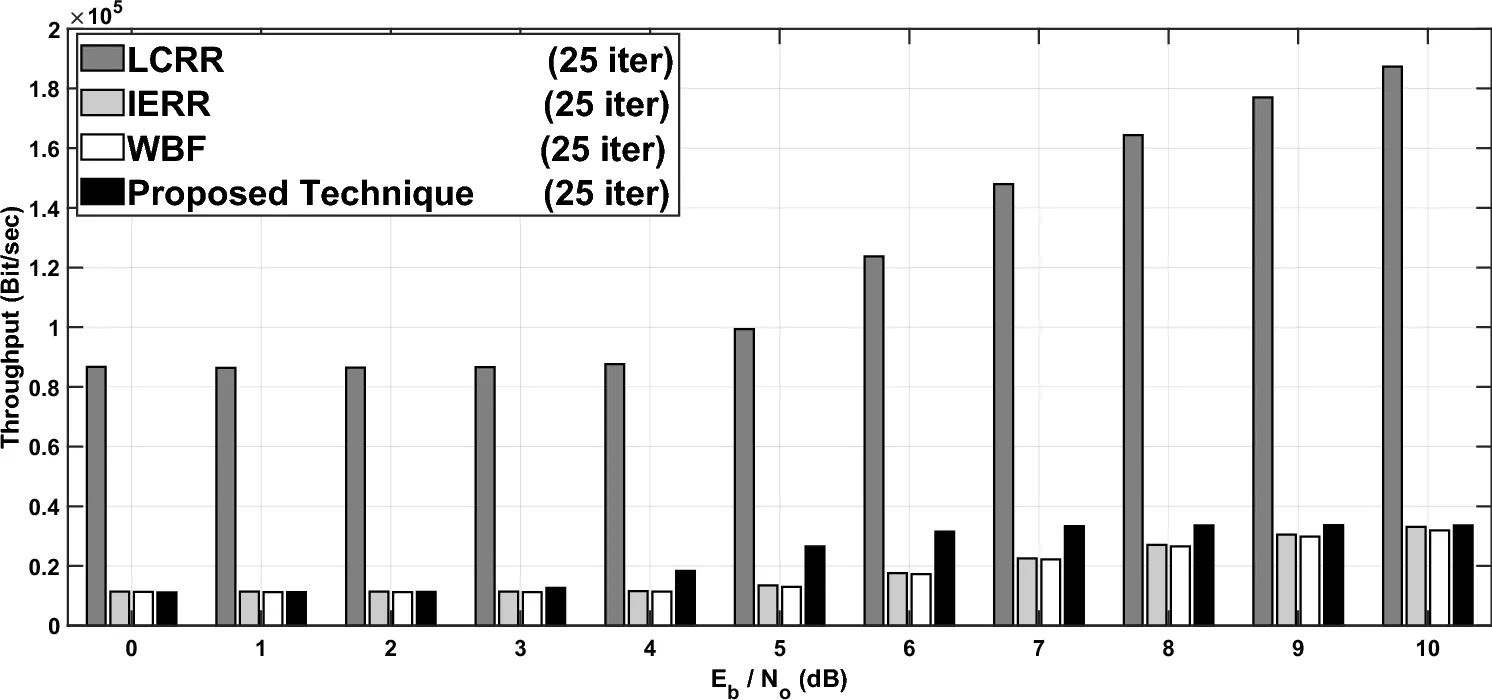
Throughput of LDPC decoders over uplink thin cirrus FSOCS communication links.

**Fig. 18:**
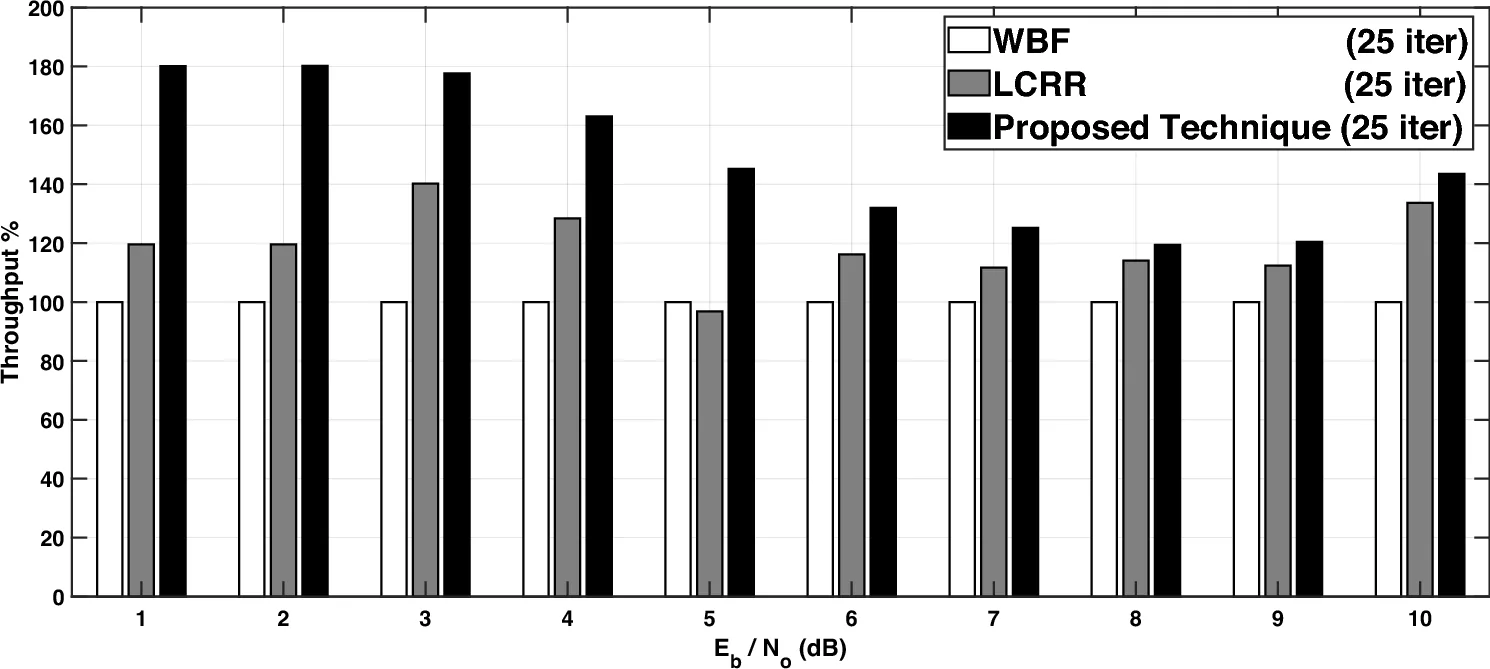
Throughput percentage of LDPC decoders over uplink thin cirrus FSOCS communication links.

The throughput and throughput percentage for the case of uplink thin cirrus communication links are shown in Figs. 17 and 18, respectively. According to Fig. 17, the proposed LDPC decoding technique achieves the highest throughput at all $E_{b} / N_{o}$, and the same performance of the proposed technique occurs in Fig. 18 in achieving the highest throughput percentage. It is observed in Figs. 17 and 18 show a variation of throughputs of all LDPC decoding techniques; this is due to the atmospheric turbulence that exists in the thin cirrus channel. Another evaluation parameter for showing the improvement imposed by the proposed decoding technique is the throughput percentage for all LDPC decoders maintained on the downlink thin cirrus communication channel. As shown in Fig. 18, the same variations occur due to atmospheric turbulence. The proposed decoding technique attains the highest throughput percentage in comparison to other decoding techniques. This assures that proposed technique success in improving the communication link effectively.

### Uplink/downlink cirrus communication channels

The simulations used in evaluating cirrus atmospheric communication links in the case of uplink and downlink are illustrated in detail as follows:

#### Uplink cirrus communication links

**Fig. 19:**
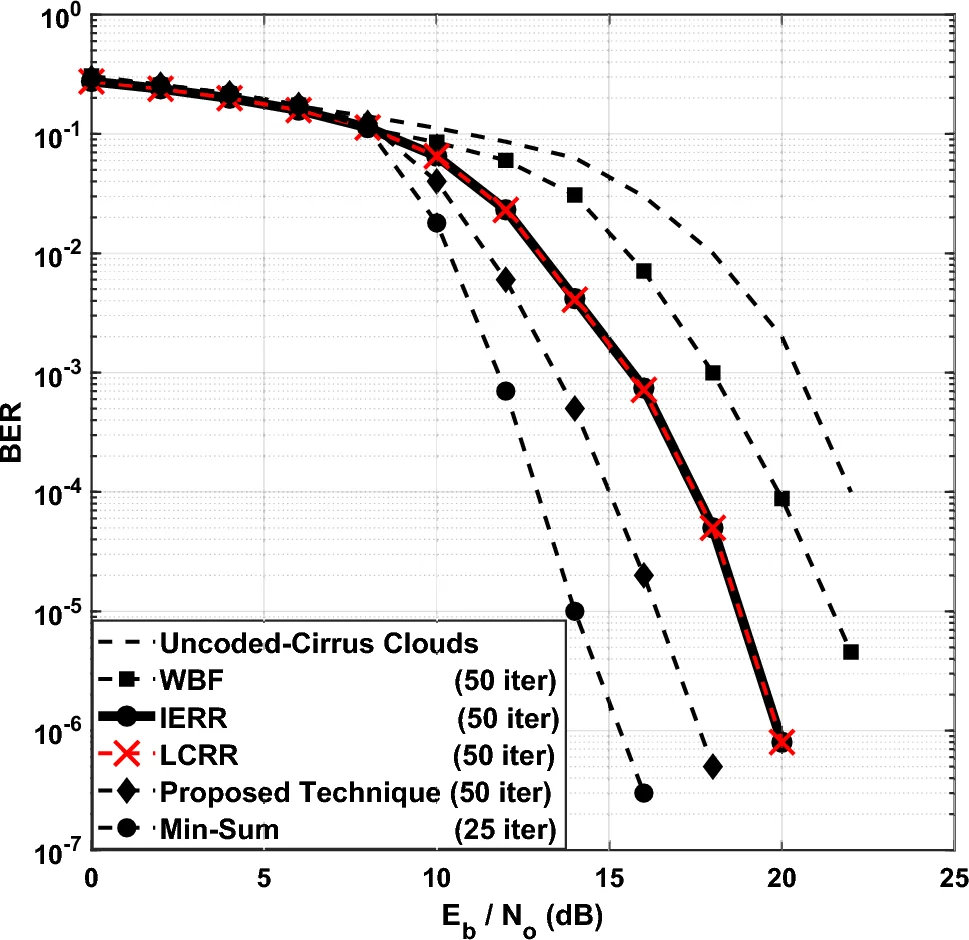
BER of LDPC decoders over uplink cirrus FSOCS communication links.

The evaluation of the impact of applying LDPC decoding techniques included the proposed decoding technique; the BER performance of the established benchmark decoders is shown in Fig. 19. According to Fig. 19, the proposed decoding technique improved the BER performance more than other LDPC decoding techniques, which demonstrates its impressive capability to mitigate severe channel impairments in the uplink Cirrus channel. In addition, the proposed decoding technique achieves at least a 2 dB improvement over the LCRR decoding technique. This improvement is due to delaying the reported oscillation that occurs in a high number of iterations used by LCRR. This causes the LCRR technique to proceed in correcting the received codeword, resulting in lower BER.

**Fig. 20:**
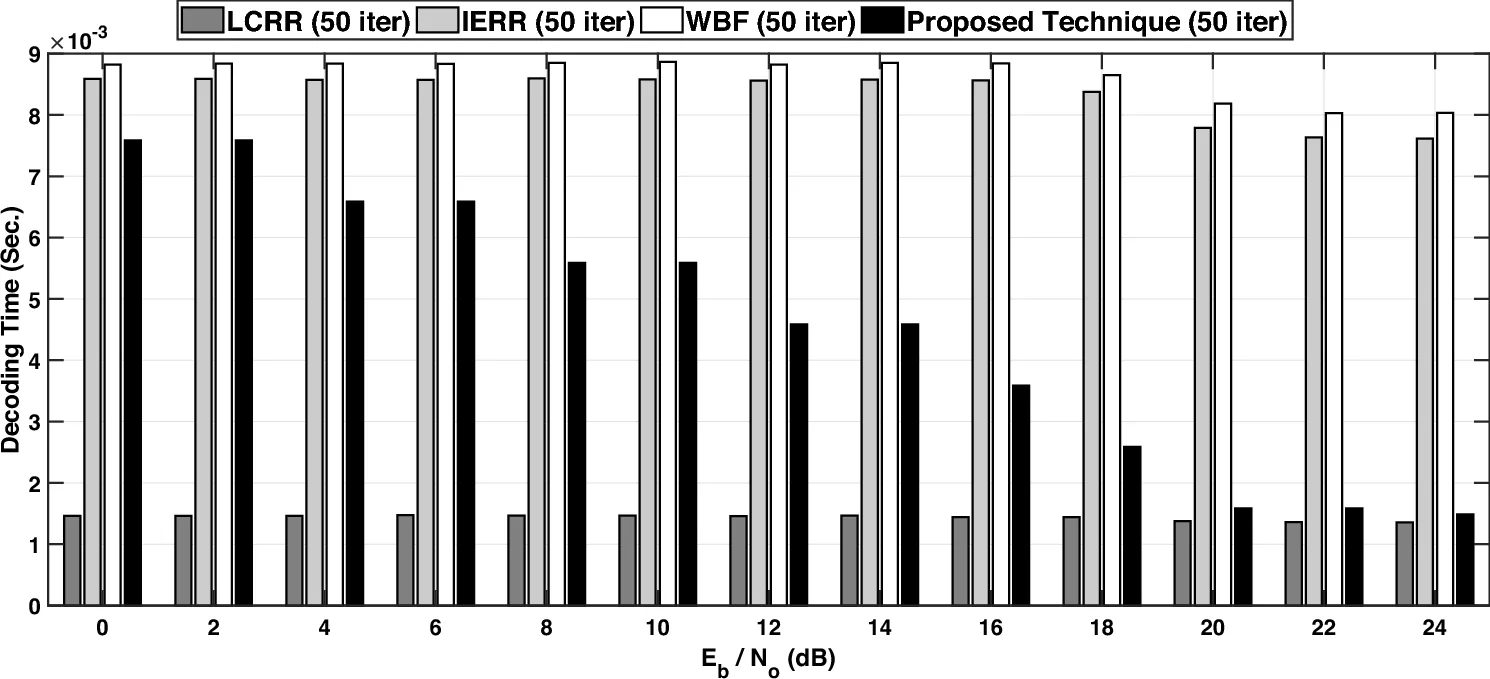
Decoding time elapsed by LDPC decoders over uplink cirrus FSOCS communication links.

The decoding time elapsed by the maintained LDPC decoding techniques is a vital parameter for evaluating those decoding techniques against the uplink Cirrus communication channel. In Fig. 20, LCRR attended the lowest decoding time in the $E_{b} / N_{o}$s range from 0 to 18 dB, while the proposed technique has the same decoding time in the $E_{b} / N_{o}$s range from 20 to 24 dB, and it succeeds in getting a lower BER compared to the LCRR technique. This confirms the ability of the proposed decoding technique to mitigate severe channel impairments without wasting excessive iterations.

**Fig. 21:**
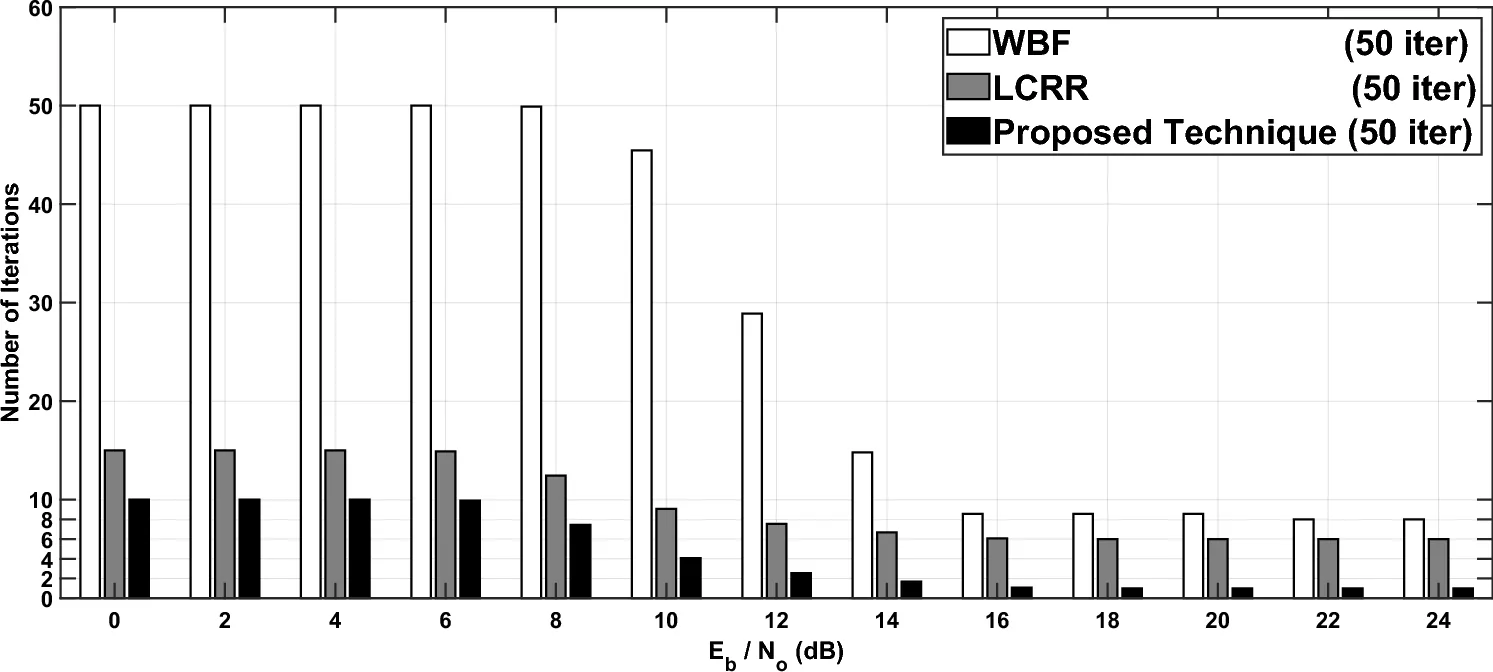
Number of iterations consumed by LDPC decoders over uplink cirrus FSOCS communication links.

In Fig. 21, the proposed decoding technique consumed the lowest number of iterations compared to all decoding techniques at all $E_{b} / N_{o}$s. This ensures that the proposed decoding technique has better performance while the maintained channel has severe impairments. Also, this improvement is because the effect of the initialization step added by the proposed decoding technique delays and reduces the oscillation phenomenon that occurs in the LCRR decoding technique.

**Fig. 22:**
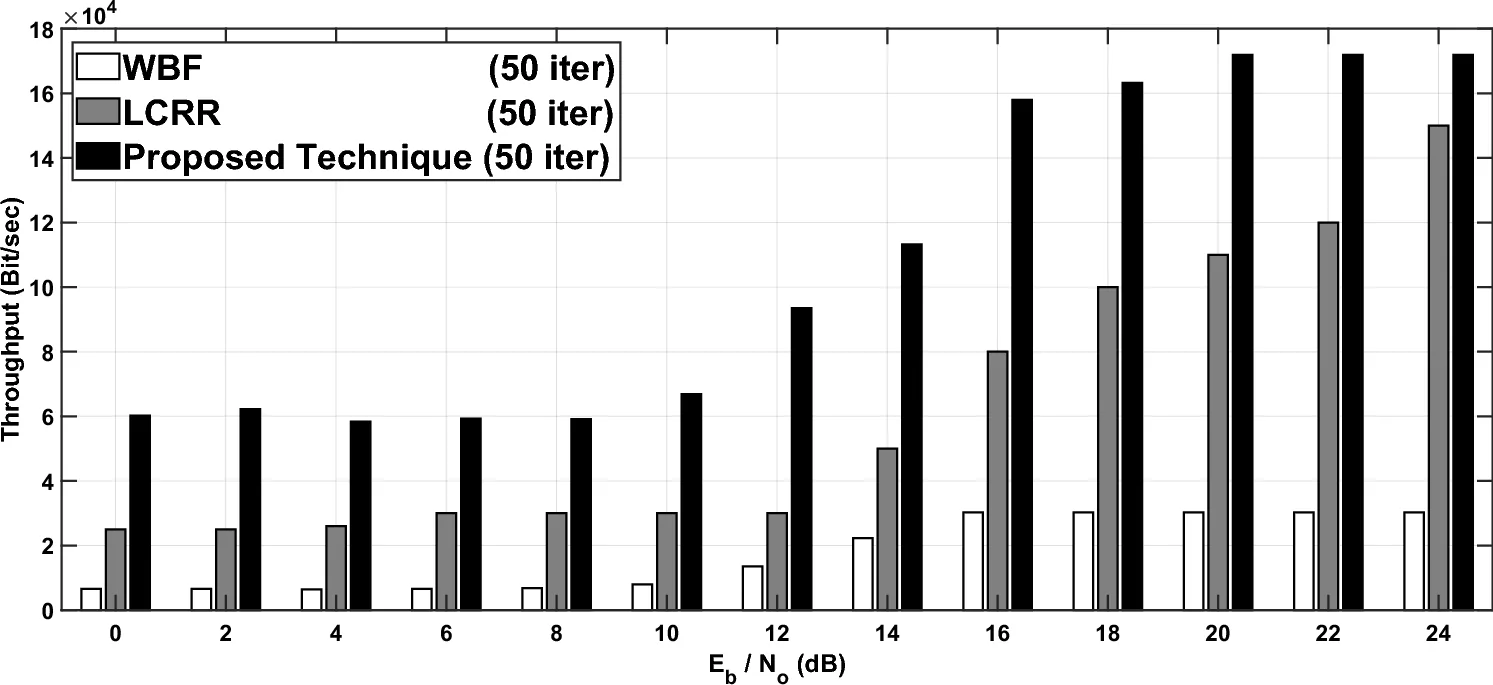
Throughput of LDPC decoders over uplink cirrus FSOCS communication links.

**Fig. 23:**
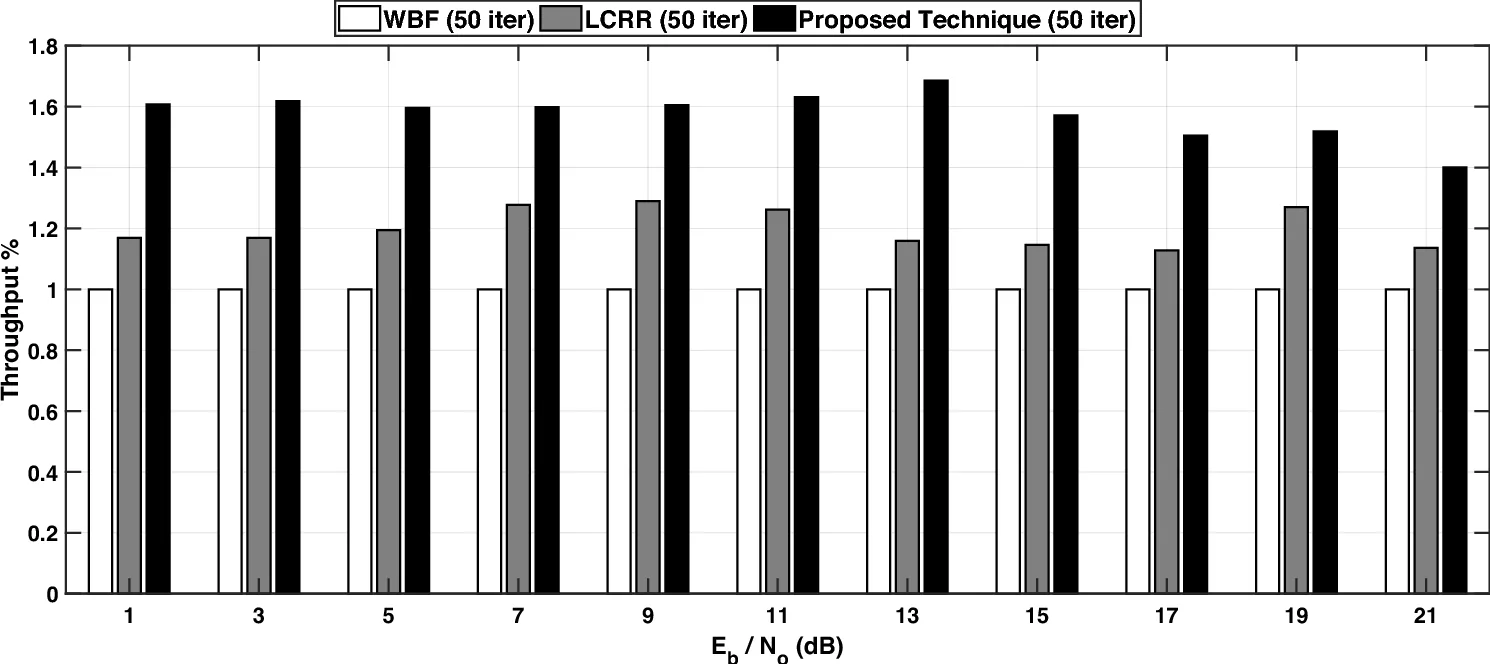
Throughput percentage of LDPC decoders over uplink cirrus FSOCS communication links.

The throughput resulting from the LDPC decoding techniques is a fundamental component in measuring the improvement imposed on the FSOCS communication links. The throughput of LDPC decoding techniques, accompanied by the proposed one, is presented in Fig. 22. The proposed technique results in the highest throughput in comparison to other LDPC decoders under study. This remarkable throughput was produced by the proposed decoding technique, proving its ability to improve the channel with excessive impairments.

Another parameter that concerns the throughput of the LDPC is the throughput percentage. The proposed decoding technique achieved the top throughput percentage compared to other LDPC decoding techniques, as shown in Fig. 23. According to Fig. 23, there is an oscillation for all established benchmark decoders due to the atmospheric turbulence that exists in the cirrus communication links.

**Fig. 24:**
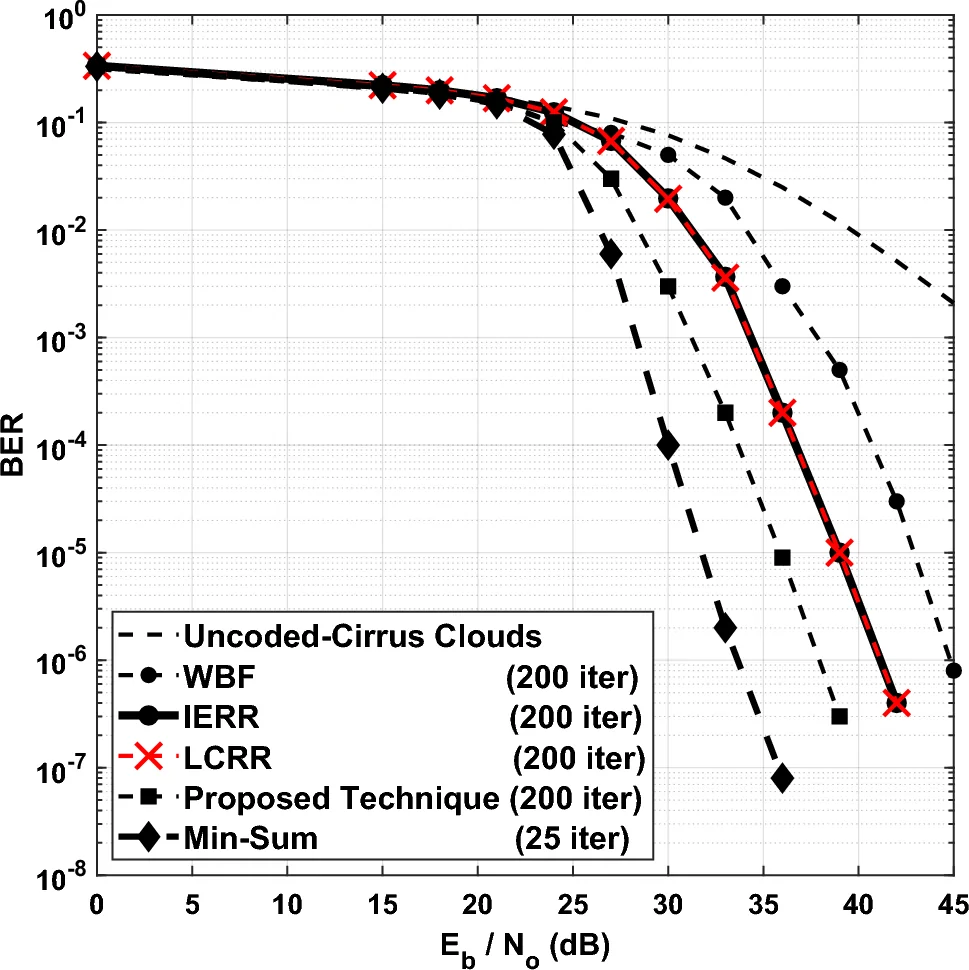
BER of LDPC decoders over downlink cirrus FSOCS communication links.

#### Downlink cirrus communication links

The LDPC decoders in this work are evaluated against downlink Cirrus communication links to perform a comprehensive evaluation of the effect imposed by the usage of LDPC decoders in the case of downlink Cirrus communication links. The BER of all LDPC decoders employed in the downlink Cirrus communication links is shown in Fig. 24. As shown in Fig. 24, the channel has excessive impairments, which require strong error control techniques to make communication possible in this case. As shown in Fig. 24, the proposed decoding technique has a lower BER compared to other decoding techniques, including LCRR. The impact of the BER performance by the proposed technique is less than on the latter channel models due to the excessive impairments in this channel.

**Fig. 25:**
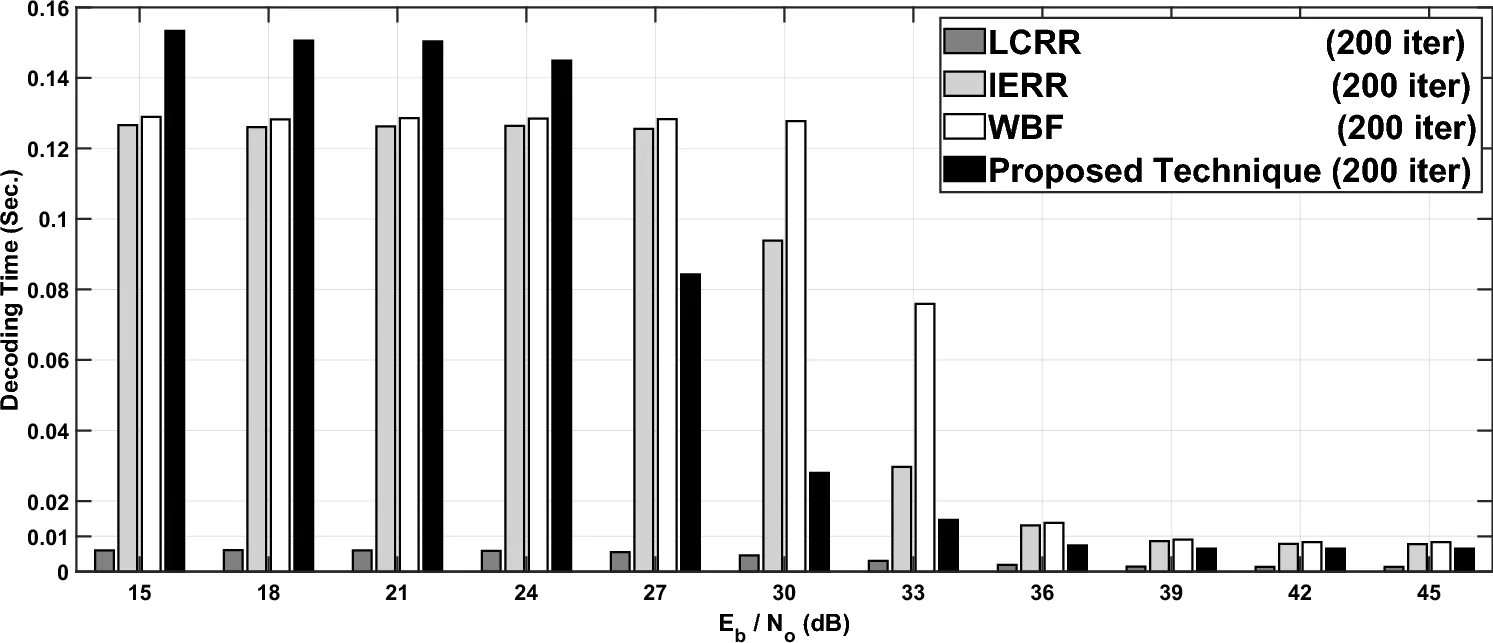
Decoding time elapsed by LDPC decoders over downlink cirrus FSOCS communication links.

The decoding time consumed by the LDPC decoding techniques to reach the BER in Fig. 24 is presented in Fig. 25. The proposed decoding technique consumed the highest decoding time, exactly at $E_{b} / N_{o}$s 16 to 24 dBs. In the higher $E_{b} / N_{o}$s from 27 to 46 dB, it approaches the decoding time of LCRR, which is characterized by very low complexity. This evaluation presents the improvement imposed by the proposed decoding technique by mitigating and lowering the severe impairments of downlink cirrus communication links with low computational processing in comparison to other LDPC decoding techniques in this study.

**Fig. 26:**
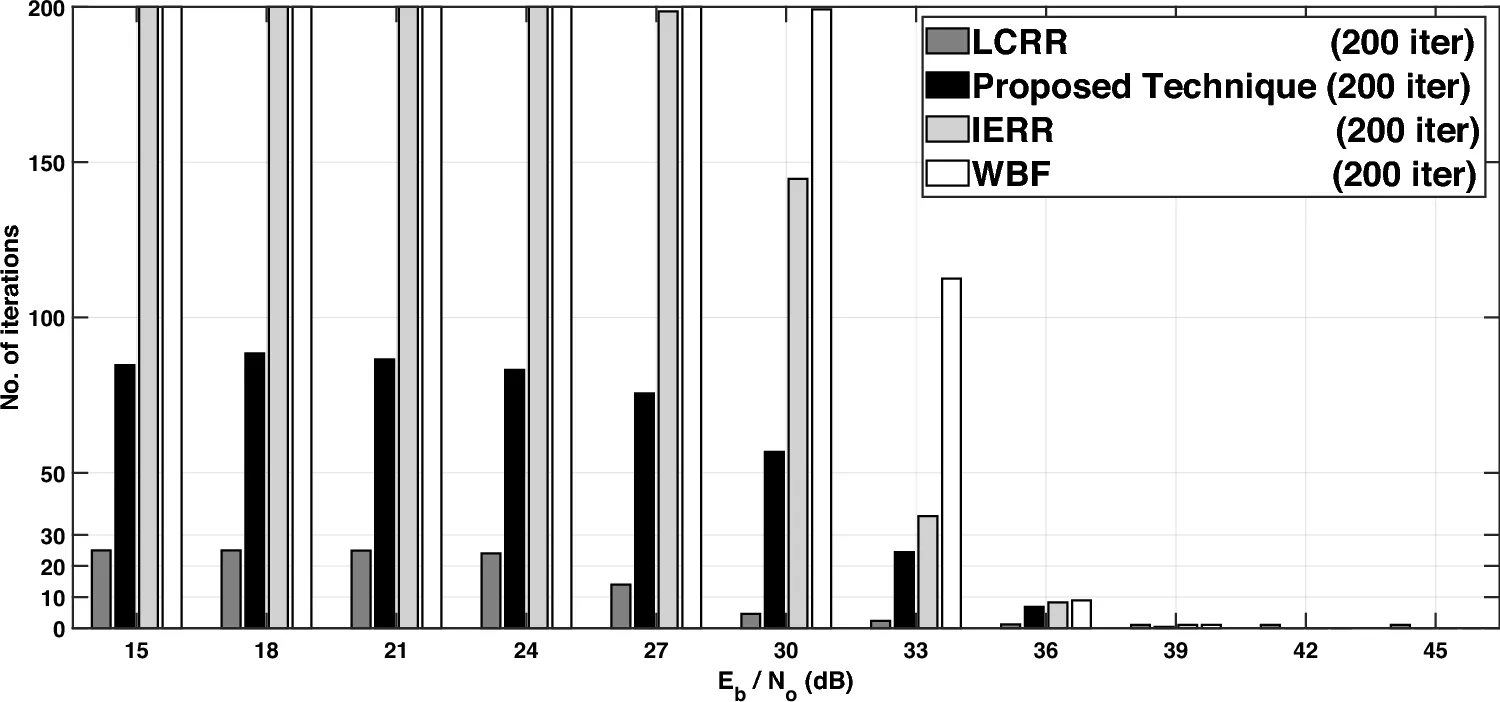
Number of iterations consumed by LDPC decoders over downlink cirrus FSOCS communication links.

Another crucial parameter in evaluating the LDPC decoders in this work is the required number of iterations to achieve the BER delineated in Fig. 24. In Fig. 26, the number of iterations required to reduce errors in received codewords of the proposed decoding technique is second place after the LCRR decoding technique. This implies that the proposed decoding technique can get a lower BER using fewer iterations in comparison to the WBF and IERR decoding techniques.

**Fig. 27:**
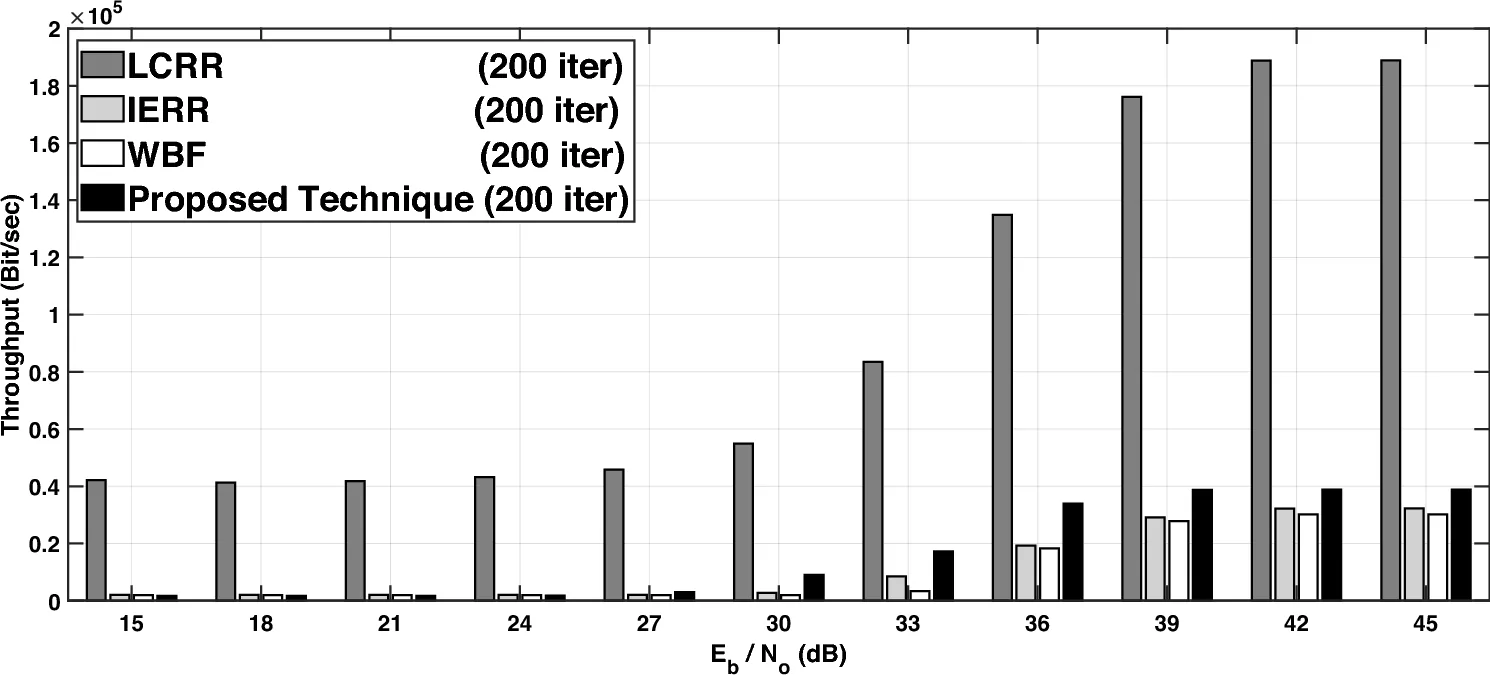
Throughput of LDPC decoders over downlink cirrus FSOCS communication links.

**Fig. 28:**
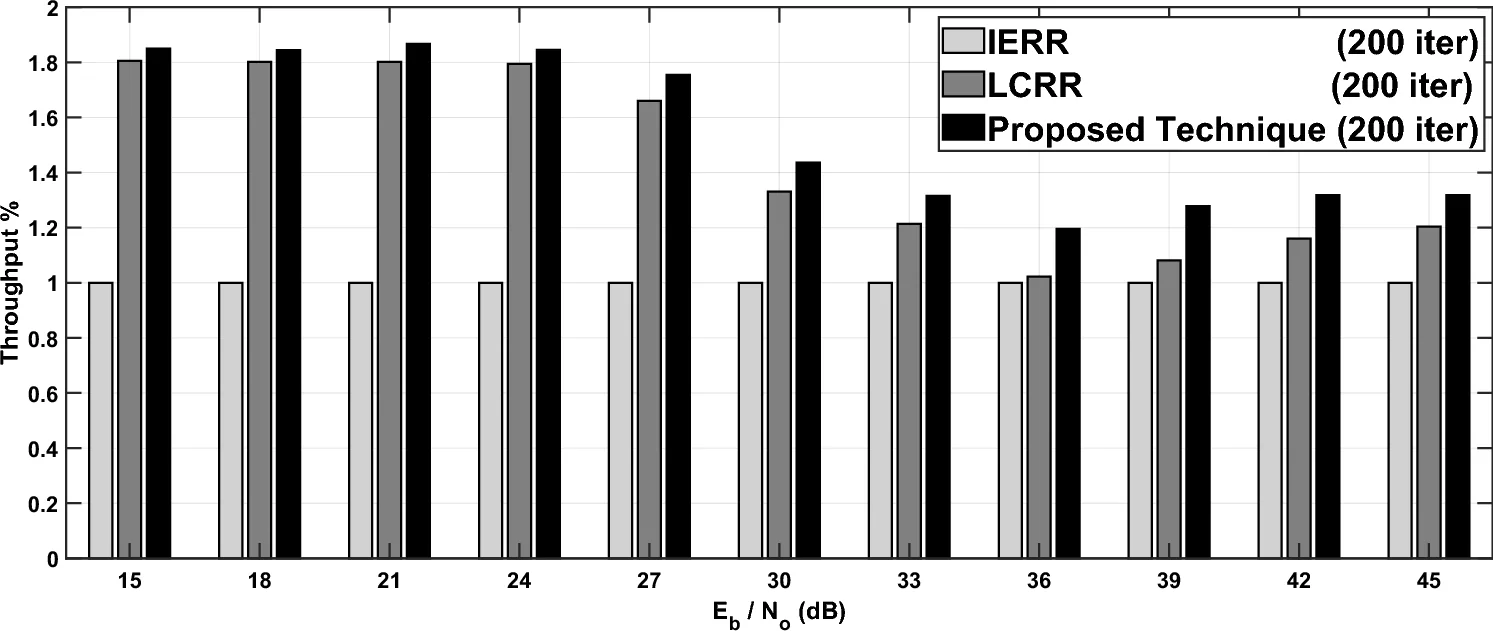
Throughput percentage of LDPC decoders over downlink cirrus FSOCS communication links.

The throughput in the case of the downlink Cirrus FSOCS communication channels is shown in Fig. 27. It is shown that LCRR has the highest throughput, while the proposed decoding technique retains the second level and the highest throughput level in comparison to other decoders under evaluation. This performance presents the improvement achieved by the proposed decoding technique in comparison to the WBF and IERR techniques. While LCRR has the best throughput, it suffers from a lower BER in comparison to the proposed decoding technique.

The proposed decoding technique achieved the highest throughput percentage, as shown in Fig. 28. There is a fluctuation in the throughput percentages across all decoding techniques, indicating the negative effect of atmospheric turbulence on the downlink Cirrus FSOCS communication links.

To provide a highly precise numerical comparison alongside the graphical curves, Table 5 presents a quantitative summary of the BER performance achieved by the evaluated decoders at selected key $E_b/N_o$ checkpoints ($E_b/N_o = 6.0 \; \text {dB}$ to $39.0 \; \text {dB}$) across representative atmospheric channels.

**Table 5:** BER comparison matrix at key SNR checkpoints.

Atmospheric condition	$E_b/N_o$	Standard WBF	IERR	LCRR	Proposed decoder
Downlink thin cirrus	6	$8 \times 10^{-2}$	$3 \times 10^{-2}$	$3 \times 10^{-2}$	$1 \times 10^{-3}$
	8	$1 \times 10^{-2}$	$2 \times 10^{-3}$	$2 \times 10^{-3}$	$2 \times 10^{-4}$
	10	$4 \times 10^{-4}$	$2 \times 10^{-5}$	$2 \times 10^{-5}$	$8 \times 10^{-7}$
Uplink thin cirrus	6	$6 \times 10^{-2}$	$2 \times 10^{-2}$	$2 \times 10^{-2}$	$5 \times 10^{-3}$
	8	$8 \times 10^{-3}$	$1 \times 10^{-3}$	$1 \times 10^{-3}$	$7 \times 10^{-5}$
	10	$2 \times 10^{-4}$	$1 \times 10^{-4}$	$1 \times 10^{-4}$	$5 \times 10^{-7}$
Uplink cirrus	10	$2 \times 10^{-2}$	$6 \times 10^{-2}$	$6 \times 10^{-2}$	$4 \times 10^{-2}$
	15	$1.8 \times 10^{-2}$	$2 \times 10^{-3}$	$2 \times 10^{-3}$	$1 \times 10^{-4}$
	18	$1 \times 10^{-3}$	$5 \times 10^{-5}$	$5 \times 10^{-5}$	$5 \times 10^{-7}$
Downlink cirrus	30	$5 \times 10^{-2}$	$2 \times 10^{-2}$	$2 \times 10^{-2}$	$3 \times 10^{-3}$
	35	$4 \times 10^{-3}$	$4 \times 10^{-4}$	$4 \times 10^{-4}$	$2 \times 10^{-5}$
	39	$2 \times 10^{-3}$	$2 \times 10^{-5}$	$2 \times 10^{-5}$	$3 \times 10^{-7}$

### Decoding time per iteration for all FSOCS cirrus types

**Fig. 29:**
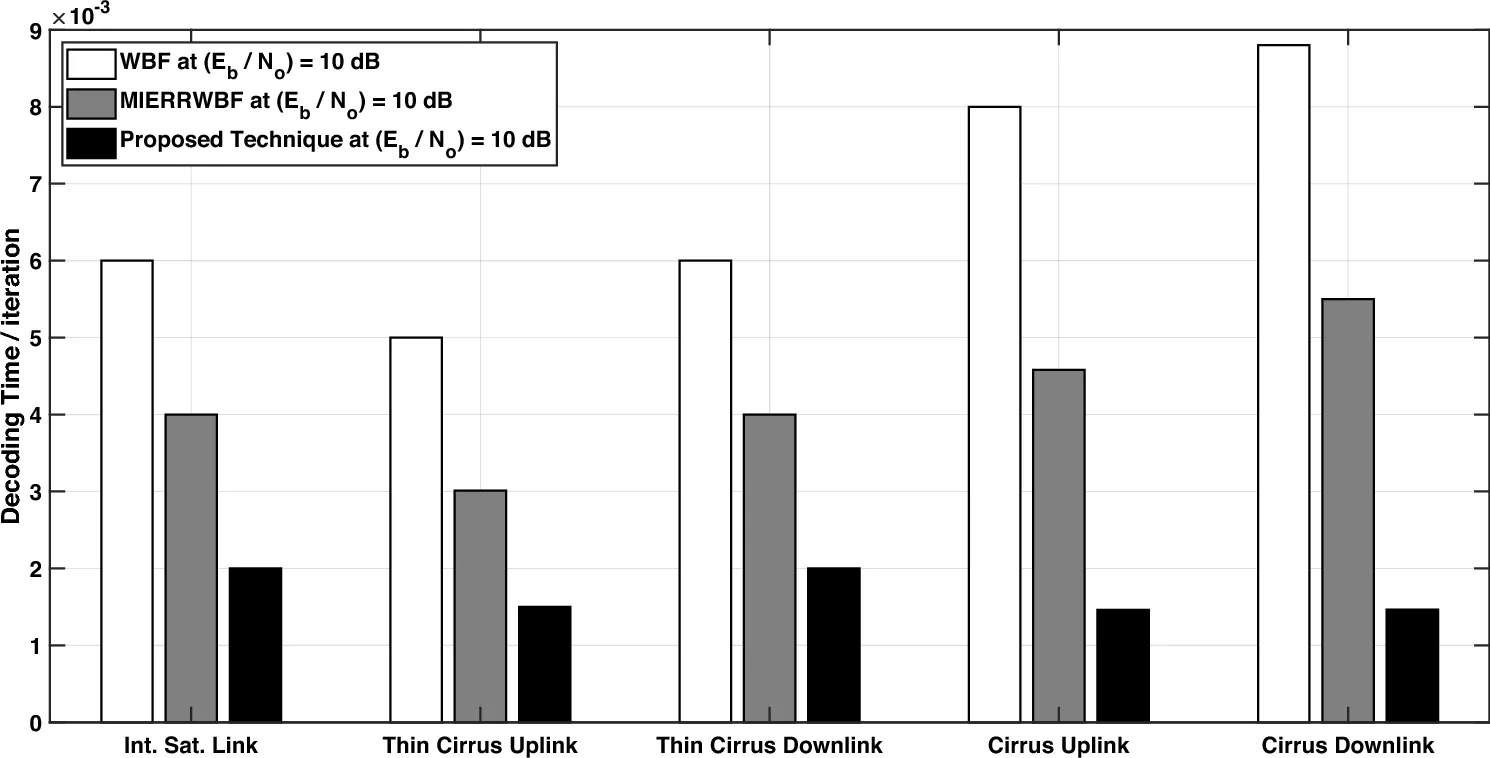
Decoding time per iteration for all FSOCS communication links.

In this part of the evaluation, all LDPC decoding techniques introduced in this work, including the proposed one, are evaluated by determining the decoding time per iteration. In Fig. 29, the proposed decoding technique achieves the lowest decoding time per iteration. This evaluation shows how much the proposed decoding technique improves communication in all FSOCS communication links.

It is critical to observe that under certain extremely low-SNR operational regimes, the total cumulative decoding time of the proposed method may marginally exceed that of the LCRR decoder. This phenomenon is a direct artifact of LCRR’s aggressive termination criteria. LCRR utilizes a strict 3-entry cyclic shifting register to abruptly kill the decoding process the exact moment an oscillation pattern is identified, preventing further processing cycles. Conversely, our proposed approach prioritizes mathematical correction by actively delaying and mitigating these cyclic error roots to systematically converge toward a correct codeword. Consequently, at very low SNRs where corruption is dense, our decoder performs extra iterations to completely rectify blocks that LCRR simply discards via early termination. However, as the channel moves into mid-to-high SNR bounds, the total latency of our scheme drops sharply below LCRR, as fewer corrective steps are triggered.

The practical impact of the architectural differences highlighted in “Proposed low-complexity LDPC decoding scheme” (Table 1) is empirically validated by the execution timings shown in Figure 31. While LCRR achieves early termination under specific conditions, its structural loop-checking overhead results in higher latency per individual cycle. Conversely, by isolating only the minimum soft value $\vert {}y_{n_{min}}\vert {} = \min (\vert {}y_n\vert {})$ per violated check node and utilizing an entirely dynamic bootstrap mechanism, our proposed decoder achieves the lowest decoding time per individual iteration layer among all evaluated hard-decision methods. This reduction in per-iteration processing complexity directly translates to the higher overall throughput percentages observed across the various communication links.

To validate the theoretical failure boundaries discussed in “Memory access, node-selection, and stopping overhead”, the decoding trajectory was tracked during dense multi-bit error events at low $E_{b}/N_{o}$s. In instances where multiple channel bits within a single check node experienced simultaneous severe fading, the algorithm initially encountered error-masking loops. However, rather than stalling indefinitely, the dynamic soft-value updates effectively broke the symmetry of the tied nodes ($\vert {}y_{n_1}\vert {} \approx \vert {}y_{n_2}\vert {}$) over 2-3 iterations, shifting the syndrome weight toward zero and achieving convergence where traditional static WBF failed.

It is explicitly noted that the execution timings logged in Fig. 29 are measured via MATLAB on a general-purpose Intel x86 CPU architecture. While absolute execution time in seconds on a desktop CPU is not a direct numerical representation of physical ASIC/FPGA hardware throughput or CubeSat onboard processing latency, it serves as a reliable relative behavioral benchmark. Because all algorithms were executed under identical system priorities, memory allocations, and software optimization flags, the fact that the proposed decoder consistently yields the lowest processing time per individual iteration layer demonstrates a fundamentally reduced computational workload. This relative efficiency directly corresponds to fewer required clock-cycle profiles when ported to dedicated embedded flight hardware.

While the proposed algorithm significantly reduces resource utilization, its boundary performance exhibits trade-offs compared to unconstrained soft-decision architectures. Specifically, under deep fading events where signal attenuation drops below the decoder’s threshold, the hard-decision quantization bounds prevent soft-bit likelihood propagation, leading to temporary frame processing delays due to iterative error-masking loops. Future work will explore dynamic threshold scaling to mitigate these localized latencies during severe turbulence fades.

## Discussion

The behavioral trends observed in the simulation results yield critical technical insights into the interaction between physical-layer atmospheric turbulence and decoder pathing. Under weak turbulence regimes, the log-amplitude variance of the FSO channel is highly stable, causing the channel LLRs to cleanly cluster away from zero. Consequently, our syndrome-driven selector isolates the single true erroneous variable node ($y_{n_{min}}$) with near-absolute certainty on the first pass. Conversely, under strong Gamma-Gamma turbulence, deep fading events collapse the received signal-to-noise ratio dynamically. This triggers multi-bit clustering errors where several adjacent variable nodes feature identically low soft values ($\vert {}y_{n_1}\vert {} \approx \vert {}y_{n_2}\vert {}$). The extended discussion in “Memory access, node-selection, and stopping overhead” highlights how our architecture successfully navigates these deep fades by leveraging the layered iterative back-end to sequentially break this soft-value symmetry across successive layers, preventing the static stagnation that causes traditional WBF to fail.

## Future work

In future multi-CubeSat operational scenarios, the proposed low-complexity decoding scheme can serve as a core physical-layer enabler for intelligent distributed networking. Because our algorithm eliminates the need for prior offline channel state estimation, it fits seamlessly into edge-computed satellite swarms that rely on real-time, adaptive cross-link coordination. Future research will explore integrating this decoder into joint resource allocation frameworks, such as adaptive token/KV-cache selection architectures, where physical-layer throughput metrics can dynamically dictate network-level media scheduling and routing policies across the constellation.

## Methods

All simulation results are obtained using the MATLAB platform.

## Data Availability

The datasets used and/or analysed during the current study available from the corresponding author on reasonable request.

## Code availability

The MATLAB source code and simulation algorithms developed in this study are permanently archived and publicly available on Zenodo at https://doi.org/10.5281/zenodo.1234567^35^.
